# Coexisting commensurate and incommensurate charge ordered phases in CoO

**DOI:** 10.1038/s41598-021-98739-6

**Published:** 2021-09-30

**Authors:** Devendra Negi, Deobrat Singh, Rajeev Ahuja, Peter A. van Aken

**Affiliations:** 1 Stuttgart Center for Electron Microscopy, Max Planck Institute for Solid State Research, Heisenbergstr.1, 70569 Stuttgart, Germany; 2 Condensed Matter Theory Group, Materials Theory Division, Department of Physics and Astronomy, Uppsala University, Box 516, 75120 Uppsala, Sweden; 3 Department of Physics, Indian Institute of Technology Ropar, Rupnagar, Punjab 140001 India

**Keywords:** Materials science, Physics

## Abstract

The subtle interplay of strong electronic correlations in a distorted crystal lattice often leads to the evolution of novel emergent functionalities in the strongly correlated materials (SCM). Here, we unravel such unprecedented commensurate (COM) and incommensurate (ICOM) charge ordered (CO) phases at room temperature in a simple transition-metal mono-oxide, namely CoO. The electron diffraction pattern unveils a COM ($q_{1}$=$\frac{1}{2}(1,1,{\bar{1}})$ and ICOM ($q_{2}=0.213(1,1,{\bar{1}})$) periodic lattice distortion. Transmission electron microscopy (TEM) captures unidirectional and bidirectional stripe patterns of charge density modulations. The widespread phase singularities in the phase-field of the order parameter (OP) affirms the abundant topological disorder. Using, density functional theory (DFT) calculations, we demystify the underlying electronic mechanism. The DFT study shows that a cation disordering ($\mathrm {Co}_{1-\textit{x}}\mathrm {O}, \text {with }{} \textit{x} = 4.17 \%$) stabilizes Jahn-Teller (JT) distortion and localized aliovalent $\mathrm {Co}^{3+}$ states in CoO. Therefore, the lattice distortion accompanied with mixed valence states ($\mathrm {Co}^{3+}, \mathrm {Co}^{2+}$) states introduces CO in CoO. Our findings offer an electronic paradigm to engineer CO to exploit the associated electronic functionalities in widely available transition-metal mono-oxides.

## Introduction

Strongly correlated materials (SCM) are a long-standing and active research frontier of material science^1^. The inherent strong electronic correlations with charge, spin, lattice and orbital degrees of freedom, places SCM in the array of appealing quantum materials, and thus revives a keen interest in SCM^2–4^. The spatial symmetry of the charge distribution plays a crucial role in dictating the electronic properties of SCM^5–9^. However, the inextricable relation of lattice distortion and emergent electronic properties remains enigmatic in SCM^10–15^. Moreover, the coexisting and competing ordering of the lattice distortions also governs important electronic property e.g. superconductivity^16,17^. Therefore, understanding the interplay of compositional modulation in SCM is crucial for manipulating the important electronic properties. CO exemplifies uniquely the interplay of perturbed lattice periodicity and strong charge-lattice coupling. In a CO transition the distorted lattice modulates commensurately or incommensurately. Interestingly, such lattice instabilities demonstrate a close proximity with various technologically important electronic properties, e.g. superconductivity, giant-magnetoresistance (GMR), charge-density-waves (CDW), and metal-insulator-transition (MIT) etc.^18–22^. Therefore, engineering CO in widely available SCM can allow to exploit the broad spectrum of associated electronic functionalities. The CO-driven structural instabilities can be controlled by various external stimuli, e.g. temperature, pressure, magnetic field, non-stoichiometry, etc.^23–26^. However, to design CO, a controlled manipulation of the functional ionic defects emphasized a plausible route in SCM^27^. CO is commonly exhibited by few complex stoichiometric and non-stoichiometric mixed-valence transition-metal oxides (TMOs) compounds. Few rare examples of simple stoichiometric TMO, exhibits the CO under thermal stimuli, e.g. $\mathrm {Fe}_{3}\mathrm {O}_{4}$^28^. Among TMOs cobalatates host a fertile avenue for CO. The interplay of strong electronic correlation and the flexible charge, spin, and lattice degree of freedom of Co ion introduces CO in non-stoichiometric or doped cobalatates. Moreover, even simple stoichiometric CoO have shown promising potential for inducing CO^29^. The CO cobaltates shows various technological important electronic properties^30^. Therefore, it is desirable to exploit the cobaltates by engineering CO even in the simple stoichiometric cobaltates.

In the present investigation, we bridge this gap and demonstrate a route for inducing CO in an example rock-salt structured transition-metal mono-oxide at room temperature. We chose CoO as a test bed material due to its proven capability of inducing CO. Our TEM-based investigations unveil microscopic evidence of CO in real and reciprocal space. Further, using DFT theory calculations the underlying electronic mechanism is uncovered. Our findings highlight the potential of functional cation disordering, which can be engineer to manipulate the CO in various unexplored similar TMOs.

## Results and discussions

### Probing lattice distortions in reciprocal space

**Figure 1:**
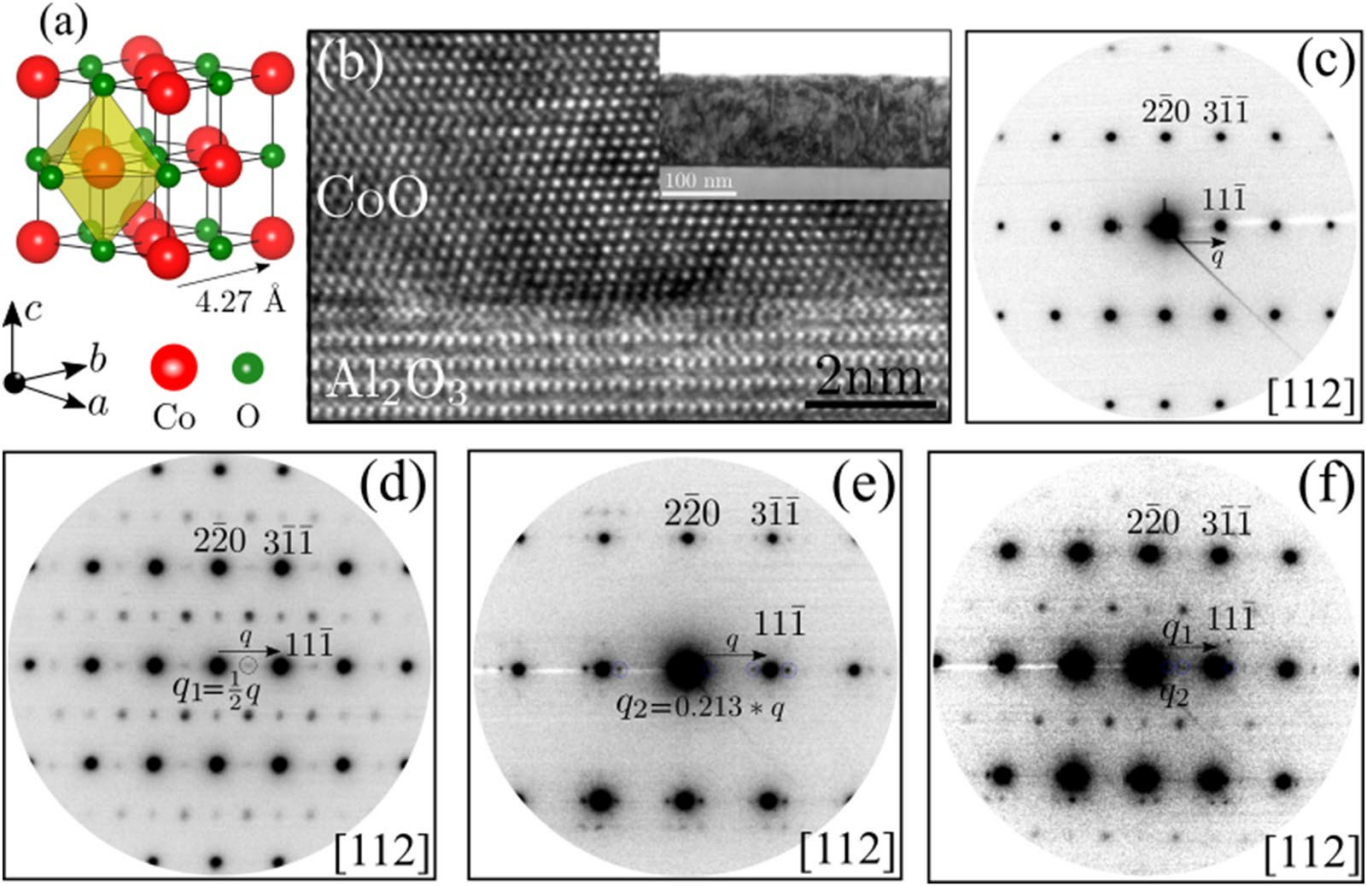
Probing the periodic lattice deformation in the reciprocal space. (**a**) Rock-salt type crystal structure of CoO. (**b**) HR-TEM image of CoO showing the interface with the sapphire substrate. The inset image shows a large-scale bright-field image of the CoO thin film. (**c**) EDP showing the crystalline nature of the CoO thin film. (**d**,**e**) EDPs showing the presence of a COM and an ICOM CO phase in the CoO lattice. (**f**) EDP showing the coexisting COM and ICOM CO phases in the CoO lattice.

CoO crystallizes in a NaCl-type rock-salt structure with the cubic space-group of $Fm{\bar{3m}}$. It is an antiferromagnetic (AFM) insulator with a lattice constant of $\sim \, 4.27$ Å (Fig. 1a)^31^. Below the Neel temperature $T_N \approx 290$ K, the magnetic moments of Co atoms stack along the (111) crystal plane. Such magnetic ordering commonly refereed as AFM-II ordering^32^. The HR-TEM image of a CoO thin film shows the epitaxial nature of the thin film (Fig. 1b). The EDP further affirms single crystallinity of the thin film (Fig. 1c). Surprisingly, the EDPs acquired from the distinct regions in the CoO thin film readily indicate the coexisting electronic phase inhomogeneity (Fig.1d–f). The apparent additional spatial frequencies in the EDP explicitly evidence the periodic lattice deformation and CO in the CoO lattice^33,34^. The quantified spatial frequencies reveal a COM (${\mathrm {q}_1} =\frac{1}{2}(1,1,{\bar{1}})$ and ICOM (${\mathrm {q}_2}=(0.213(1,1,{\bar{1}}))$) (Fig. 1d,e) CO phase. However, in some regions in the thin film, the EDP also suggests the coexisting superlattices (Fig.1 f). The coexisting nature of the COM and ICOM superlattice phases in a SCM also reported^23,35^. The EDP also suggest the compositional modulation in the thin film. The theory of spinodal-decomposition can provide an additional insight on the free-energy associated to the compositional-modulation to justify the stability of such coexisting CO phases in CoO^36^. However, this study lies beyond the scope of the present investigation and is reserved for future investigations. Nevertheless, the present experimental observation inspires to explore the aspects of a spinodal-decomposition for CoO and for other similar TMOs. Furthermore, a cohesive energy analysis indicates that at higher temperatures cation vacancies can easily form in CoO (“First principles calculations”) Such a vacancy can form at a temperature of (900$^{\circ }$) during thin-film growth^37^. Although an anion vacancy can also be formed, the ordering of cation vacancies is energetically more preferable then anion-vacancy ordering^38^. Moreover, a cation deficiency in CoO implies the formation of mixed-valence charge states (“First principles calculations”, Fig. S15). The mixed-valence states accompanied with a lattice distortion offer the prospect of CO (Supplementary Information). Therefore, the superlattice spots in the EDP represent a cation-disordering-induced CO in CoO. The underlying electronic mechanism of CO is elaborated in “First principles calculations”. The occurrence of COM and ICOM CO phase at room temperature in CoO is a unique observation and suggests a yet unexplored prospect in such simple rock-salt structured transition-metal mono-oxides. The capability of tailoring such composition modulation by controlled defect engineering in the widely available TMOs offers potential opportunities for leveraging electronic properties associated to the CO.

### Microscopic structural features of CO

**Figure 2:**
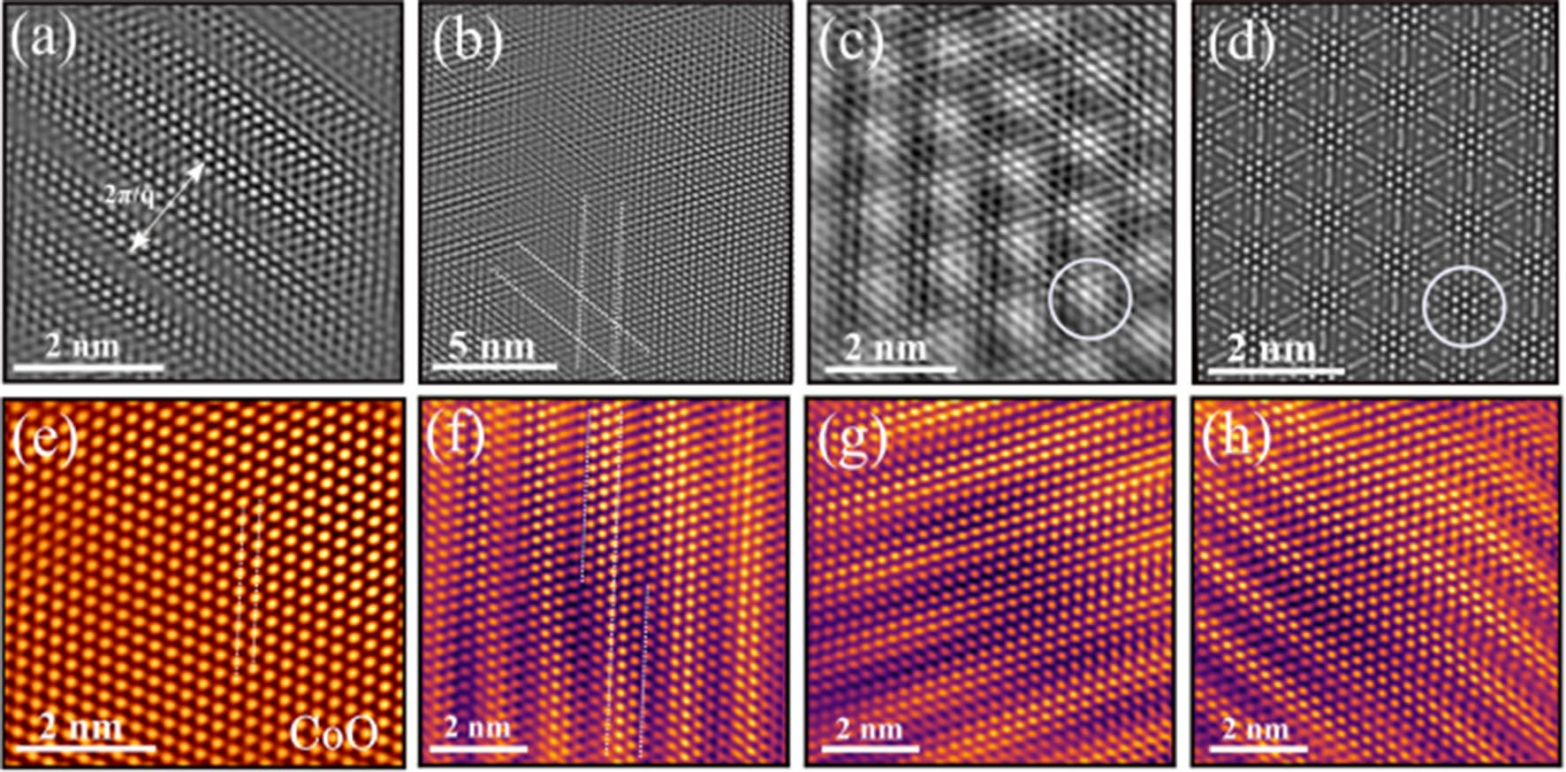
Real-space distribution of local electronic-phase inhomogeneity in CoO. (**a**,**b**) Unidirectional and bidirectional stripe patterns of the charge density modulation in CoO. (**c**) Intensity modulation of a segregated charge density in CoO. (**d**) Simulated HR-TEM image of $\mathrm {Co}_{1-\textit{x}}\mathrm {O}$ shows a close similarity with (**c**) the experimental HR-TEM. The circles indicate the equivalent location of a segregated density in the experimental and simulated HR-TEM image, respectively. (**e**) HR-TEM image of the unperturbed lattice of CoO. (**f**–**h**) Bending and breaking nature of CO domains.

The Fourier filtered electron micrograph of COM phase (${q_1}$) displays a unidirectional charge density modulation along the [112] zone-axis (Fig. 2a). These unidirectional density modulations are referred as charge-stripe pattern. Such stripe pattern are recognized as a characteristic microscopic structural features of a CO material^39^. These unidirectional charge density stripes also serve as a real-space evidence of broken translational and rotational symmetry in a CO transition^40^ (Supplementary Information, Fig. S2). In contrast, the ICOM phase shows bidirectional charge density modulations (Fig. 2b). These density stripes mutually intertwine. The checkerboard-type CO materials also exhibits a similar stripe patterns^40–43^ (Supplementary Information, Fig. S3). Furthermore, a detailed microscopic inspection allows us to witness a region, which exhibits an intriguing pattern of segregated charge density modulations. A similar nature of charge density modulation has been reported for various CDW materials^44–46^. The segregated charge density presumably manifest the highly localized Coulomb interaction^47^. A previous theoretical study has established that a cation imperfection in CoO leads to a charge-state disproportions^48^. Therefore, a broken spatial symmetry and mixed-valence charge-states poses a prospect of CO in $\mathrm {Co}_{1-{\textit{x}}}\mathrm {O}$. We align our investigation with such insights and explore the feasibility of CO in $\mathrm {Co}_{1-{\textit{x}}}\mathrm {O}$. The potential of such an assumption is substantiated by simulating the HR-TEM image of $\mathrm {Co}_{1-{\textit{x}}}\mathrm {O}$ at 300 kV TEM. The non-stochiometric crystal structure constructed by creating a Co vacancy at the center of a ($2 \times 2 \times 2$) supercell of CoO. The simulated HR-TEM images displays a qualitatively resemblance with the experimental image. The simulated HR-TEM demonstrates the exact segregated charge density modulation pattern at corresponding locations (Fig. 2c,d). A qualitative similarity and a quantitative comparison of the experimental and simulated micrographs consolidates the aforementioned assumption of cation disordering (Supplementary Information, Fig. S15). Next, we explore the spatial uniformity of the CO domains, along different crystal plane ((${\bar{{2}}00}$), (${\bar{{1}}1{\bar{1}}}$), (${11{\bar{1}}}$)) directions along the [011] zone-axis. The unperturbed CoO lattice shows a regular atomic modulation (Fig. 2e). However, the CO domains demonstrate a wrinkled nature of the atomic modulation. The bending and breaking nature of CO modulation can be radially identified (Fig. 2f–h). The bending and breaking nature of density modulation signifies the dislocations in the modulation of CO domains. A similar bending and breaking nature of CO domains also reported recently in a complex CO magnetite^49^. Our previous study also traced the CO in CoO^29^. However, the present detailed investigation unveils the unexplored rich microscopic structural features of CO and further provides an invaluable insight on the relative dominance nature of such CO phase (“Spatial distribution of order parameter”). Furthermore, an extensive theoretical analysis provides a deeper understanding on the operating electronic mechanism, and elaborates the previously unnoticed JT distortion and orbital reconstruction next to vacancy site (“First principles calculations”).

### Spatial distribution of order parameter

**Figure 3:**
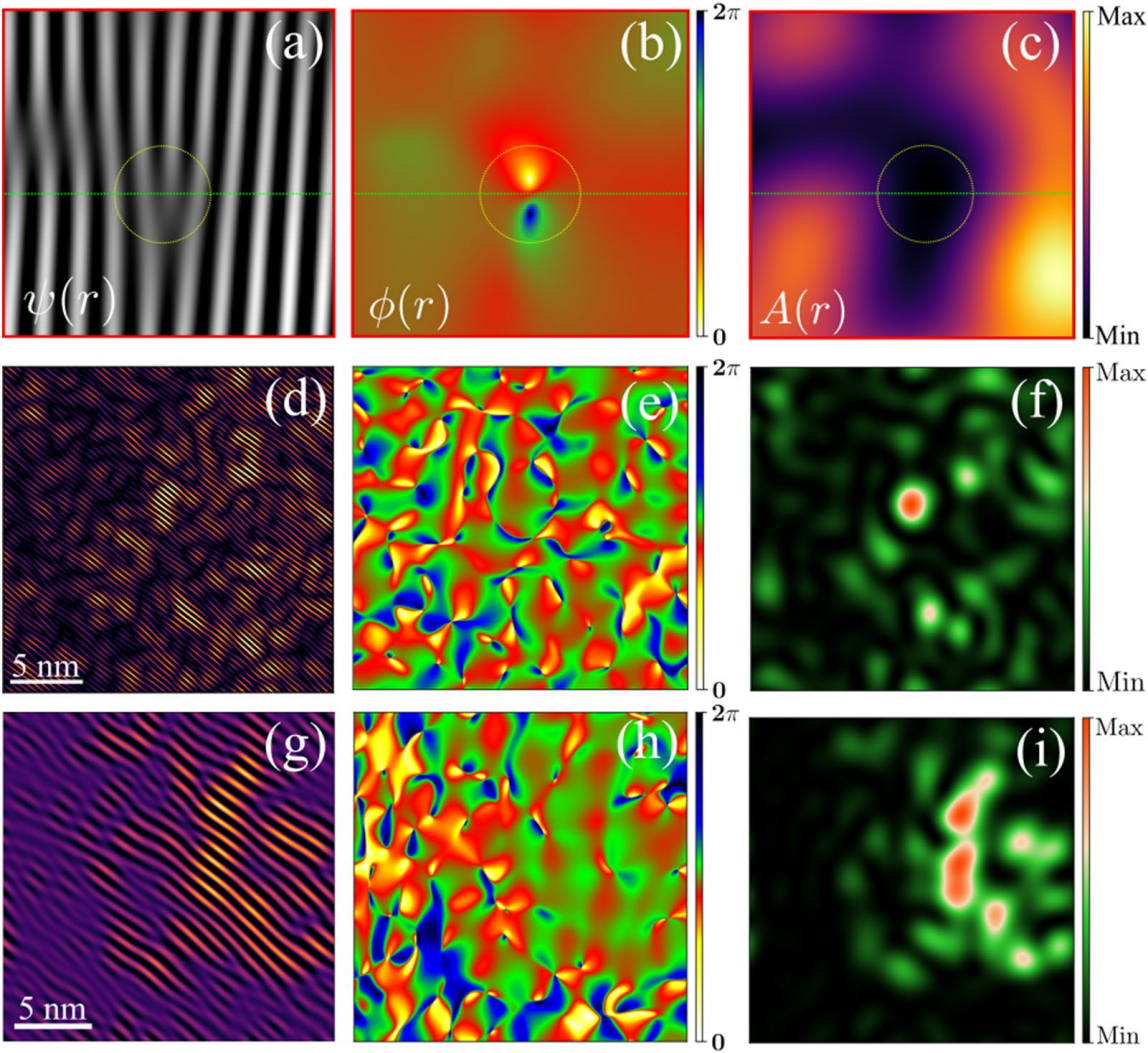
Exemplifying the coarse grain phase mapping process. (**a**–**c**) Complex field $\psi _{q}(\Delta r)$ of a unidirectional density modulation and the corresponding spatial map of ${\phi (r)}$, $\mathrm {A(r)}$, respectively. The circles mark the corresponding exact equivalent location. (**d**,**g**) Unidirectional Density modulation map of COM and ICOM phase. (**e**,**h**) Spatial ${\phi (r)}$ fluctuation map of COM and ICOM phase. (**f**,**i**) Spatial $\mathrm {A(r)}$ fluctuation map of COM and ICOM lattice modulation.

The order parameter is an important measure to gauge the degrees of order in the phase transition process. Therefore, we compute the spatial fluctuation map of the OP by using the phase-lock method^49,50^(Supplementary Information, Section II). The OP associated to the complex-field of the modulation can be approximated by the equation; ${\psi _{\mathrm {q}}(\Delta \mathrm {r}) \approx \mathfrak {R}\{\mathrm {A(r)} * {\mathrm {exp}}^{\textit{i}(\mathrm {q}\cdot {\text {r}} + \phi (\mathrm {r}))} \}}$, where $\mathrm {A(r)}$ is an displacement amplitude of the modulation. The vector *q* is a modulation vector of the superlattice reflection and ${\phi (r)}$ represents the phase-field of the modulation. ${\phi (r)}$ also encapsulates the information of the localized disorder, i.e. emergent topological defects. Topological defects are unavoidable emergent excitation, which originate during the structural phase transition process. The topological disorder can be perceived in the form of a phase singularity in ${\phi (r)}$, which winds ${2\pi }$ around the core of the topological defect. These phase singularities are similar to the quantum fluxoid and vortices in superconductors^51^ (Fig. 3b). These topological defects cost a finite energy, which contributes to stabilize the COM phase^6^. Therefore, the phase-map provides an insight on the relative spatial stability of such CO phase. The $\mathrm {A(r)}$ vanishes at the core of the localized topological defect. The phase mapping is an effective approach, which qualitatively shows the relative dominating nature of the coexisting electronic phases^6^ (Supplementary Information). Figure 3a–c, exemplify the extraction of the ${\phi (r)}$ and $\mathrm {A(r)}$ fields of a complex-field associated to the unidirectional lattice modulation. A topological discontinuity in the OP is identifiable by a ${2\pi }$ winding phase singularity in ${\phi (r)}$. Consistently, the computed spatial $\mathrm {A(r)}$ field precisely locate the minimum intensity at the corresponding location of the phase singularity and the core of topological disorder. The COM phase with ${q_1}$ exhibits an abundance of dislocations in the unidirectional density modulation (Fig. 3d,g). The corresponding ${\phi (r)}$ map shows an interconnected network of phase-singularities (Supplementary Information). The COM phase possesses more phase singularities than the coexisting ICOM CO phase (Fig. 3e,h), signifying the large lattice-order disruption in the COM order. The intensity of $\mathrm {A(r)}$ vanishes at a locations of the core of phase singularities (Fig. 3f,i). The spatial phase-fluctuation maps indicate a relative higher lattice order degradation of the COM phase. Moreover, a thermodynamic study of such CO can also firmly establish the competing nature of such coexisting CO phase^23,33^.

### First principles calculations

**Figure 4:**
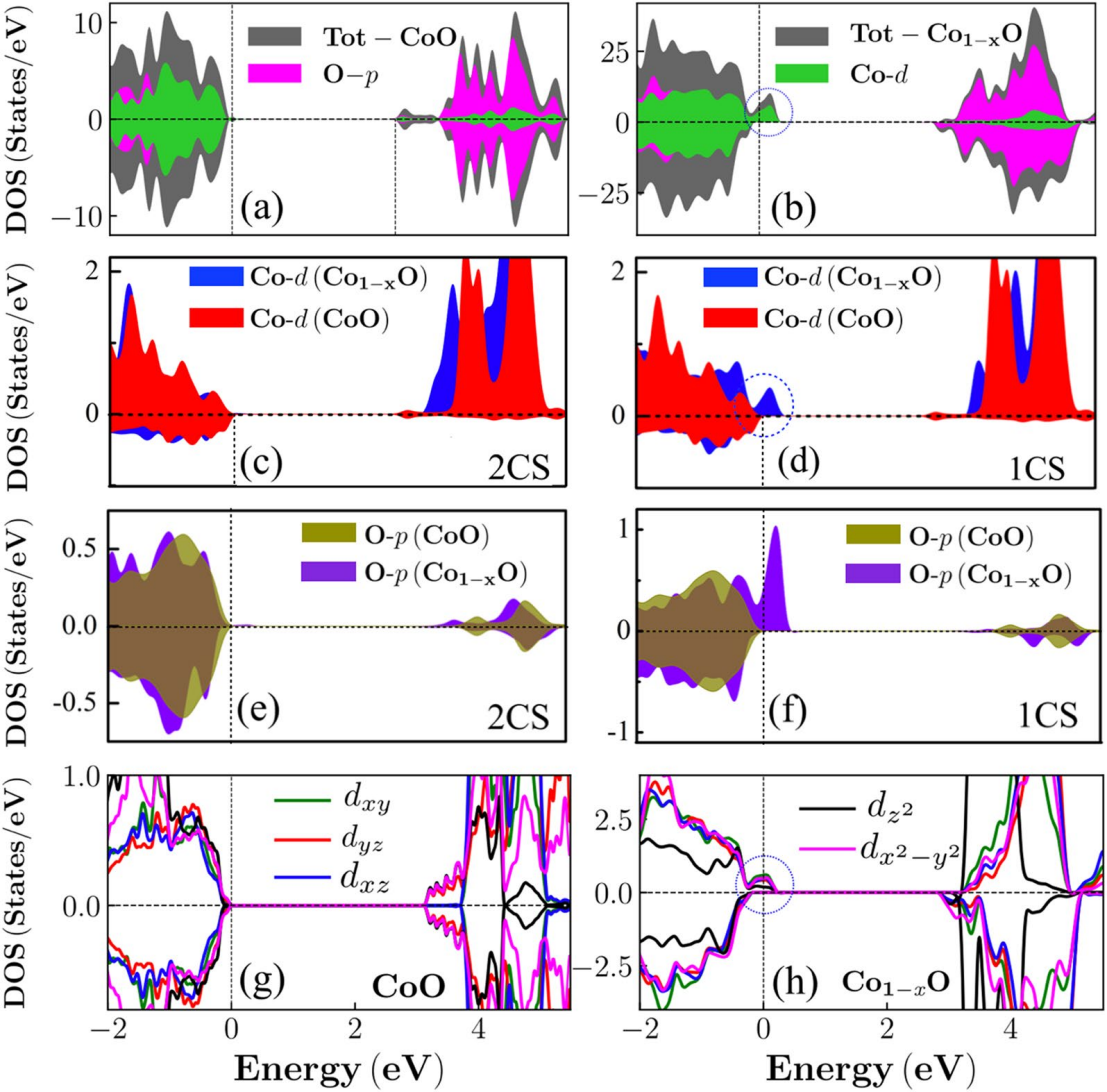
DFT calculations exploring the microscopic origin of CO in non-stoichiometric CoO. (**a**,**b**) Total DOS of CoO and $\mathrm {Co}_{1-\textit{x}}\mathrm {O}$, $x= 4.17\%$, with the contribution of Co-*d* and O-*p* states, respectively. (**c**,**d**) Comparison of the Co-*d* DOS of the first and second coordination shell (1CS, 2CS) from the $\mathrm {V}_{\mathrm {Co}}$ in $\mathrm {Co}_{1-\textit{x}}\mathrm {O}$ to the ones in CoO. (**e**,**f**) Comparison of the O-*p* DOS of the first and second coordination shell from the $\mathrm {V}_{\mathrm {Co}}$ in $\mathrm {Co}_{1-\textit{x}}\mathrm {O}$ to the ones in CoO. (**g**,**h**) Co-*d* orbital-decomposed spin-polarized DOS of CoO and $\mathrm {Co}_{1-\textit{x}}\mathrm {O}$, respectively.

Our TEM-based investigations have evidently proved the CO in real and reciprocal space, respectively. We further computationally pursue to uncover the underpinning electronic mechanism, which can justify the observed unconventional CO in CoO. It is well established that a functional ionic deficiency profoundly modifies the local structural, chemical and magnetic environment in transition-metal mono-oxides^27^. Such electronic modifications can induce diverse electronic properties, e.g. half-metallicity, spin-blockade, spin-state crossover, ferromagnetism, etc, even in a simple stoichiometric transition-metal mono-oxides, e.g. CaO, MnO, NiO, CoO^38,48,52–55^. Therefore, motivated by an exceptional agreement between our experimental and simulated HR-TEM observations, and further guided by the earlier theoretical insights, we follow the route of cation order imperfection and computationally explore the feasibility of inducing CO in $\mathrm {Co}_{1-\textit{x}}\mathrm {O}$ (Supplementary Information, Section III). First, we ascertain the feasibility of inducing cation disordering in CoO based on energetic stability. A small cohesive energy ($E_c$) difference ($\Delta E_{c}\sim 0.07$ eV) between CoO ($E_{c}=4.93$ eV) and $\mathrm {Co}_{1-\textit{x}}\mathrm {O},x=4.17\%$ ($E_{c}=$ 4.86 eV) affirms that a cation vacancy can easily form during high temperature ($\sim 900^{\circ }$) film growth by pulsed laser deposition^37^. Although, the anion vacancy can be easily created at such a temperature, a cation-vacancy ordering is energetically preferable over anion-vacancy ordering^38^. Moreover, a stable non-stochiometric TMO crystal can even possess a much higher defect density than the representative minimal defect density considered in present theoretical investigations^52,56^.

CoO is a charge-transfer insulator with a band gap of $\sim 2.7$ eV. Our calculation results show an agreement with previous investigation^57^. The hybridized nature of Co-3$d-$O-2*p* states in CoO is evident from the DOS (Fig. 4a). The complementary populated spin-up and spin-down states signify the AFM ordering in CoO. The profound influence of $\mathrm {V}_{\mathrm {Co}}$ in $\mathrm {Co}_{1-\textit{x}}\mathrm {O}$ is distinguishable at Fermi level ($E_F$). The energetically emerged hybridized Co-3$d-$O-2*p* states surpass $E_F$. Interestingly, only a unidirectional spin-channel, i.e. the spin-up channel, shows the metallic nature (Fig. 4b). Such electronic states are commonly referred as half-metallic (HM) states and are considered to be extremely important for various spintronics applications. Cation defect-induced HM is also reported in other similar transition-metal mono-oxides, e.g. NiO, MnO, CaO^38,52,53,55^. The asymmetric nature of the DOS profile implies a deviation from the AFM nature. We further appraise the origin of the half-metallic state by analyzing the DOS of Co-*d* and O-*p* adjacent to and at a distance from the disorder atomic site. The DOS unequivocally demonstrates that a Co and O atoms adjacent to $\mathrm {V}_{\mathrm {Co}}$ exclusively contributes the HM states (Fig. 4c–f). Further, to gain a deeper insight on the orbital reconstruction at $E_F$, the orbital decomposed DOS is evaluated. The valence band maximum of CoO is populated by nearly uniform contribution of all Co-*d* partial orbital states (Fig. 4g). However, in $\mathrm {Co}_{1-\textit{x}}\mathrm {O}$, the energetic distribution of the partial orbitals substantially alters and surpass $E_F$. The energetic orbital states introduces the half-metallic states in $\mathrm {Co}_{1-\textit{x}}\mathrm {O}$ (Fig. 4h). The energetically emerged states signify an extended orbitals overlapping and thus prone to destabilize the crystal-field environment. The modified orbital states at disorder site reflects a perturbed structural, magnetic, and chemical environment. The hole compensation and the electrostatic interaction between defect site and loosely bound holes elongate the defected $O_h$ to reduce the in-plane (lateral) Co–O bond from 2.11 Å  to 1.95 Å  at two nearest $O_h$. However, the $O_h$ elongation concurrently increase the Co–O bond in out-of-plane (axial) direction by $\sim$ 0.10 Å. The perturbed Co–O bond lengths concomitantly distort the $O_h$ symmetry. The $O_h$ distortion can modify the chemical nature of the ionic states therefore, we gauge the chemical state by computing the ionic charges by performing Bader charge analysis^58^. The computed ionic charges on pure CoO are $q_{Co}= +1.34$ $e^{-}$ and $q_{O}= -1.34$ $e^{-}$, respectively. However, in $\mathrm {Co}_{1-\textit{x}}\mathrm {O}$, we identify higher ionic charges $q'_{Co}= +1.50$ $e^{-}$ at vertically inverted and adjacent to disorder sites. The enhanced ionic charge indicates a localized charge-state transition. Interestingly, the chemical identity of two $\mathrm {V}_{\mathrm {Co}}$-adjacent O atoms persists nearly identical to the O atom of pure CoO, i.e. $q'_{O} = -1.36$ $e^{-}$. The chemical identity remains preserved by the charge transfer from the elevated charge state. The charge transfer essentially compensates the hole formation and leads to an charge state disproportion by introducing localized aliovalent $\mathrm {Co}^{3+}$ state. The $\mathrm {V}_{\mathrm {Co}}$-induced $\mathrm {Co}^{3+}$ state is also supported by some earlier theoretical investigations^48^. Moreover, we also experimentally validated the mix-valence Co states in $\mathrm {Co}_{1-\textit{x}}\mathrm {O}$, by electron energy-loss spectroscopy (Supplementary Information, Section I(D)). The localized structural and chemical distortion simultaneously influence the corresponding magnetic nature. We identify that the spin magnetic moment of two Co atoms enhances from initial 2.75 to 3.15 $\upmu_{B}$. These two Co atoms reside vertically opposite from the cation deficient atomic site, undergo a higher charge-state transition and reduce the bonding distance. Moreover, the uplifted magnetic frustration also induces a minute moment of 0.21 $\upmu_{B}$ on two $\mathrm {V}_{\mathrm {Co}}$-adjacent O atoms. However, the Co and O atoms located far from the $\mathrm {V}_{\mathrm {Co}}$ remain nearly unmodified.

The computational insights enables to infer that a localized cation deficiency forms two uncompensated hole states in the O-*p* bands. These holes are compensated by a charge delocalization from partially occupied Co-*d* states, located vertically opposite to the disordered atomic site. The charge-transfer process transforms the divalent Co ion to a trivalent state ($\mathrm {Co}^{3+}$). The hole-compensation process also displaces the cation from a high symmetry location in $O_h$ geometry. Therefore, lowering the symmetry activates a JT distortion (Supplementary Information). In $\mathrm {Co}_{1-\textit{x}}\mathrm {O}$, the JT distortion elongates the cation-deficient octahedra ($O_{h}^{V_{Co}}$). The axial elongation of $O_{h}^{V_{Co}}$ reduces the symmetry in adjacent compressed octahedra ($O_{h}^{c}$) and uplift the degeneracy via destabilizing the energy of the $d_{xy}$, $d_{x^{2}-y^{2} }$ orbitals (Fig. 4h). However, the $O_h$ elongation concomitantly stretches $O_h$ in lateral direction (Supplementary Information). The $O_h$ compression also facilitates to eliminate the degeneracy of the crystal-field ($\Delta _{octa}$) by destabilizing the energy of the $d_{z^{2}}$, $d_{xz}$, $d_{yz}$ orbitals (Fig. 4h). The computed orbital-decomposed DOS capture the energetic of the crystal-field distortion and corroborates the operating JT mechanism in $\mathrm {Co}_{1-\textit{x}}\mathrm {O}$ (Fig. 4h). Therefore, the energetically destabilized partial orbital states cross $E_F$ to form the HM states. Thus, a localized cation imperfection induces a JT displacement and consequent charge-transfer introduces aliovalent states ($\mathrm {Co}^{2+}$, $\mathrm {Co}^{3+}$) (Supplementary Information, Section III(D)). Hence, the DFT calculation justify the experimental observations and suggests that a cation disordering-induced lattice distortion accompanied with mixed-valence charge states leads to the CO in $\mathrm {Co}_{1-\textit{x}}\mathrm {O}$.

## Discussion

To summarize, we unravel COM and ICOM CO phases in CoO. The TEM-based investigations show the characteristics of the CO phase in real and reciprocal space, respectively. Furthermore, the coupled DFT calculations decipher the underlying electronic mechanism. The DFT calculation highlights the cation-disorder-driven functional structural, chemical and magnetic modification, which leads to the mix-valence charge states in CoO. The lattice distortion accompanied with mix-valence states leads to the evolution of CO phases in $\mathrm {Co}_{1-\textit{x}}\mathrm {O}$. CO shows a close proximity with various important electronic properties. Therefore, our finding suggest that a controlled functional cation-ordering imperfection offers opportunities to engineer and leverage the broad spectrum of emergent functionalities, even in widely available simple transition-metal mono-oxides.

## Methods

### Thin film growth

Epitaxial thin film of CoO is grown by pulsed laser deposition method^37^. The thin film is grown on the “${{c}}$” plane of sapphire ($\mathrm {Al}_{2}\mathrm {O}_{3}$) substrate. The target pallet of CoO prepared by grinding CoO power (Sigma aldrich 99.99% purity) and further sintering it for 9 h in tube furnace. An excimer LASER (KrF) with $\lambda \sim$ 248 nm wavelength (Laser fluence $\,\sim 1.5\, \mathrm {J cm}^{-2}$) used to ablate CoO from the target pallet. The target pallet is placed $\sim 5$ cm far from the $\mathrm {Al}_{2}\mathrm {O}_{3}$ substrate. The oxygen partial pressure in the chamber is maintained at $\mathrm {P}_{\mathrm {O}_2} \sim 1\times 10^{-5}$ Torr.

### Electron microscopy characterization

Various TEM-based characterizations techniques, e.g. high-resolution TEM (HR-TEM) imaging, electron diffraction pattern (EDP), bright-field (BF), dark-field (DF) imaging and electron energy-loss spectroscopy (EELS) were performed at 300 kV in the aberration correction electron microscope FEI-TITAN (Supplementary Information, Section I).

### Density functional theory calculations

The electronic structure calculations are based on the spin-polarized density functional theory (DFT), as implemented in the vienna ab-initio simulation package (VASP)^59,60^. The exchange-correlation energy treated within the generalized gradient approximation (GGA) in the form of the Perdew, Burke and Ernzerhof (PBE) functional^61^. The plane-wave basis cut-off was set to 600 eV. The Monkhorst–Pack (MP) method with ($20 \times 20 \times 4$) *k*-points mesh selected for CoO and $\mathrm {Co}_{1-\textit{x}}\mathrm {O}$, respectively^62^. The $\mathrm {Co}_{1-\textit{x}}\mathrm {O}$ structure was constructed by embedding a Co vacancy ($\mathrm {V}_{\mathrm {Co}}$) in a ($2 \times 2 \times 2$) supercell (48 atoms) of CoO ($\mathrm {Co}_{1-{\textit{x}}}\mathrm {O}, \textit{x} = 4.17 \%$) (Supplementary Information). For structural optimizations, a combination of the conjugate-gradient algorithm and the quasi-Newton force minimization was used. The limit of the force tolerance was set to $5\times 10^{-3}$ eV/Å. The Coulomb repulsion *U* = 7.1 eV and the local exchange interactions with *J* = 1 eV, are used for the on−site interaction of Co-3*d* states^48^.

## Supplementary Information

Supplementary Information.

## References

[1] Morosan E, Natelson D, Nevidomskyy AH, Si Q (2012). Strongly correlated materials. Adv. Mater..

[2] Keimer B, Moore J (2017). The physics of quantum materials. Nat. Phys..

[3] Basov D, Averitt R, Hsieh D (2017). Towards properties on demand in quantum materials. Nat. Mater..

[4] Tokura Y, Kawasaki M, Nagaosa N (2017). Emergent functions of quantum materials. Nat. Phys..

[5] Fradkin E, Kivelson SA (2010). Electron nematic phases proliferate. Science.

[6] Mesaros A (2011). Topological defects coupling smectic modulations to intra-unit-cell nematicity in cuprates. Science.

[7] Fradkin E, Kivelson SA, Lawler MJ, Eisenstein JP, Mackenzie AP (2010). Nematic fermi fluids in condensed matter physics. Annu. Rev. Condens. Matter Phys..

[8] Lawler M (2010). Intra-unit-cell electronic nematicity of the high-$${T}_c$$ copper-oxide pseudogap states. Nature.

[9] Vojta M (2009). Lattice symmetry breaking in cuprate superconductors: Stripes, nematics, and superconductivity. Adv. Phys..

[10] Anderson PW (1972). More is different. Science.

[11] Comin R (2015). Symmetry of charge order in cuprates. Nat. Mater..

[12] Kivelson SA, Fradkin E, Emery VJ (1998). Electronic liquid-crystal phases of a doped mott insulator. Nature.

[13] Hinkov V (2008). Electronic liquid crystal state in the high-temperature superconductor $${\rm YBa_{2}Cu_{3}O_{6}}$$. Science.

[14] Kotliar G, Vollhardt D (2004). Strongly correlated materials: Insights from dynamical mean-field theory. Phys. Today.

[15] Tokura Y (2003). Correlated-electron physics in transition-metal oxides. Phys. Today.

[16] Chang J (2012). Direct observation of competition between superconductivity and charge density wave order in $${\rm YBa_{2}Cu_{3}O_{6.67}} $$. Nat. Phys..

[17] Yu Z-D, Zhou Y, Yin W-G, Lin H-Q, Gong C-D (2017). Phase competition and anomalous thermal evolution in high-temperature superconductors. Phys. Rev. B.

[18] Gao D, Liu Y, Zhao H, Mou Y, Feng S (2018). Interplay between charge order and superconductivity in cuprate superconductors. Phys. C (Amsterdam, Neth.).

[19] Rao C, Cheetham A (1997). Charge ordering in manganates. Science.

[20] Tomioka Y, Asamitsu A, Kuwahara H, Moritomo Y, Tokura Y (1996). Magnetic-field-induced metal-insulator phenomena in $${\rm Pr_{1-x}Ca_{x}MnO_{3}}$$ with controlled charge-ordering instability. Phys. Rev. B.

[21] Moritomo Y, Takeo M, Liu X, Akimoto T, Nakamura A (1998). Metal-insulator transition due to charge ordering in $${\rm R_{1/2}Ba_{1/2}CoO_{3}}$$. Phys. Rev. B.

[22] Moritomo Y, Asamitsu A, Kuwahara H, Tokura Y (1996). Giant magnetoresistance of manganese oxides with a layered perovskite structure. Nature.

[23] Chen C, Cheong S-W (1996). Commensurate to incommensurate charge ordering and its real-space images in $${\rm La_{0.5}Ca_{0.5}MnO_{3}}$$. Phys. Rev. Lett..

[24] Moritomo Y, Kuwahara H, Tomioka Y, Tokura Y (1997). Pressure effects on charge-ordering transitions in perovskite manganites. Phys. Rev. B.

[25] Wu T (2011). Magnetic-field-induced charge-stripe order in the high-temperature superconductor $${\rm YBa_{2}Cu_{3}O_{y}}$$. Nature.

[26] Rao C, Arulraj A, Cheetham A, Raveau B (2000). Charge ordering in the rare earth manganates: The experimental situation. J. Phys. Condens. Matter.

[27] Kalinin SV, Spaldin NA (2013). Functional ion defects in transition metal oxides. Science.

[28] Verwey E (1939). Electronic conduction of magnetite ($${\rm Fe_{3}O_{4}}$$) and its transition point at low temperatures. Nature.

[29] Negi D, Loukya B, Datta R (2015). Native defect induced charge and ferromagnetic spin ordering and coexisting electronic phases in coo epitaxial thin film. Appl. Phys. Lett..

[30] Raveau B, Seikh MM (2015). Charge ordering in cobalt oxides: Impact on structure, magnetic and transport properties. Z. Anorg. Allg. Chem..

[31] Van Elp, J. *et al.* Electronic structure of $${\rm CoO, Li-}$$ doped $${\rm CoO}$$ and $${\rm LiCoO_{2}}$$ (1991). 10.1103/physrevb.44.6090 9998470

[32] Roth W (1958). Magnetic structures of $${\rm MnO, FeO, CoO}$$, and $${\rm NiO}$$. Phys. Rev..

[33] Ramirez A (1996). Thermodynamic and electron diffraction signatures of charge and spin ordering in $${\rm La_{1-x}Ca_{x}MnO_{3}}$$. Phys. Rev. Lett..

[34] Zuo J-M, Tao J (2001). Nanometer-sized regions of charge ordering and charge melting in $${\rm La_{2/3}Ca_{1/3}MnO_{3}}$$ revealed by electron microdiffraction. Phys. Rev. B.

[35] Haupt K (2016). fast metamorphosis of a complex charge-density wave. Phys. Rev. Lett..

[36] Binder K (1987). Theory of first-order phase transitions. Rep. Prog. Phys..

[37] Loukya B (2011). Controlling structural quality of $${\rm ZnO}$$ thin film on $$c$$-plane sapphire during pulsed laser deposition. J. Cryst. Growth.

[38] Park S (2008). Interaction and ordering of vacancy defects in $${\rm NiO}$$. Phys. Rev. B.

[39] Mori S, Chen C, Cheong S-W (1998). Pairing of charge-ordered stripes in $${\rm (La, Ca)MnO_{3}}$$. Nature.

[40] Comin R (2015). Broken translational and rotational symmetry via charge stripe order in underdoped $${\rm YaBa_{2}Cu_{3}O_{6+y}}$$. Science.

[41] Kang M (2019). Evolution of charge order topology across a magnetic phase transition in cuprate superconductors. Nat. Phys..

[42] Li Z (2009). A “checkerboard” orbital-stripe phase and charge ordering transitions in $${\rm Pr(Sr_{x}Ca_{2-x})Mn_{2}O_{7}} (0<x<0.45)$$. Europhys. Lett..

[43] Zheng Q (2018). Real space visualization of competing phases in $${\rm La_{0.6}Sr_{2.4}Mn_{2}O_{7}}$$ single crystals. Chem. Mater..

[44] Lutsyk I (2018). Electronic structure of commensurate, nearly commensurate, and incommensurate phases of $$\rm 1T-TaS_{2}$$ by angle-resolved photoelectron spectroscopy, scanning tunneling spectroscopy, and density functional theory. Phys. Rev. B.

[45] Pásztor Á (2019). Holographic imaging of the complex charge density wave order parameter. Phys. Rev. Res..

[46] Pásztor Á, Scarfato A, Barreteau C, Giannini E, Renner C (2017). Dimensional crossover of the charge density wave transition in thin exfoliated $${\rm VSe_{2}}$$. 2D Mater..

[47] Ekvall I, Kim J-J, Olin H (1997). Atomic and electronic structures of the two different layers in $${\rm 4Hb-TaS_{2}}$$ at 4.2 k. Phys. Rev. B.

[48] Wdowik U, Parlinski K (2008). Electronic structure of cation-deficient $${\rm CoO}$$ from first principles. Phys. Rev. B.

[49] Savitzky BH (2017). Bending and breaking of stripes in a charge ordered manganite. Nat. Commun..

[50] El Baggari I (2018). Nature and evolution of incommensurate charge order in manganites visualized with cryogenic scanning transmission electron microscopy. Proc. Natl. Acad. Sci..

[51] Tilley DR (2019). Superfluidity and Superconductivity.

[52] Osorio-Guillén J, Lany S, Barabash SV, Zunger A (2007). Nonstoichiometry as a source of magnetism in otherwise nonmagnetic oxides: Magnetically interacting cation vacancies and their percolation. Phys. Rev. B.

[53] Ködderitzsch D, Hergert W, Szotek Z, Temmerman W (2003). Vacancy-induced half-metallicity in $${\rm MnO}$$ and $${\rm NiO}$$. Phys. Rev. B.

[54] Negi DS, Singh D, van Aken PA, Ahuja R (2019). Spin-entropy induced thermopower and spin-blockade effect in $${\rm CoO}$$. Phys. Rev. B.

[55] Negi DS, Datta R, Rusz J (2019). Defect driven spin state transition and the existence of half-metallicity in $${\rm CoO}$$. J. Phys. Condens. Matter.

[56] Wdowik UD, Piekarz P, Parlinski K, Oleś AM, Korecki J (2013). Strong effects of cation vacancies on the electronic and dynamical properties of FeO. Phys. Rev. B.

[57] Wdowik UD, Parlinski K (2007). Lattice dynamics of $${\rm CoO}$$ from first principles. Phys. Rev. B.

[58] Tang W, Sanville E, Henkelman G (2009). A grid-based bader analysis algorithm without lattice bias. J. Phys. Condens. Matter.

[59] Parr RG (1980). Density functional theory of atoms and molecules. Horizons of Quantum Chemistry.

[60] Kresse G, Hafner J (1993). Ab initio molecular for liquid metals. Phys. Rev. B.

[61] Perdew JP, Burke K, Ernzerhof M (1996). Generalized gradient approximation made simple. Phys. Rev. Lett..

[62] Monkhorst HJ, Pack JD (1976). Special points for brillouin-zone integrations. Phys. Rev. B.

