# Supplementary material for: Coexisting commensurate and incommensurate charge ordered phases in CoO
Source: Sci Rep. 2021 Sep 30;11:19415. doi: 10.1038/s41598-021-98739-6 (PMC8484683; doi:10.1038/s41598-021-98739-6)
Supplement: Supplementary file 1 — Supplementary Information. [file 41598_2021_98739_MOESM1_ESM.pdf]

Supplementary Information

Coexisting commensurate and incommensurate charge ordered phases in CoO

Devendra Negi,[1] Deobrat Singh,[2] Rajeev Ahuja,[2,3] and Peter A. van Aken[1]

[1]*Stuttgart Center for Electron Microscopy, Max Planck Institute for Solid State Research, Heisenbergstr.1, 70569 Stuttgart, Germany*

[2]*Condensed Matter Theory Group, Materials Theory Division, Department of Physics and Astronomy, Uppsala University, Box 516, 75120 Uppsala, Sweden*

[3]*Department of Physics, Indian Institute of Technology Ropar, Rupnagar 140001, Punjab, India*

(Dated: September 4, 2021)

I. STRUCTURAL CHARACTERIZATION OF COO THIN FILM

A. Dark-field image of CO phase

This section explores the spatial characteristics of CO phase in the real-space. Figure S1 shows a dark-field (DF) image of the ICOM (q_2) CO phase. The DF image has been captured by placing the objective aperture at (111) crystal plane of the ICOM lattice. The DF image shows a wrinkle-like pattern in the thin film, which probably reflect the spatial deviation, arising from the ICOM lattice distortion. A previous investigation have also evidenced similar characteristics wavy pattern in the DF image of ICOM CO phase [1].

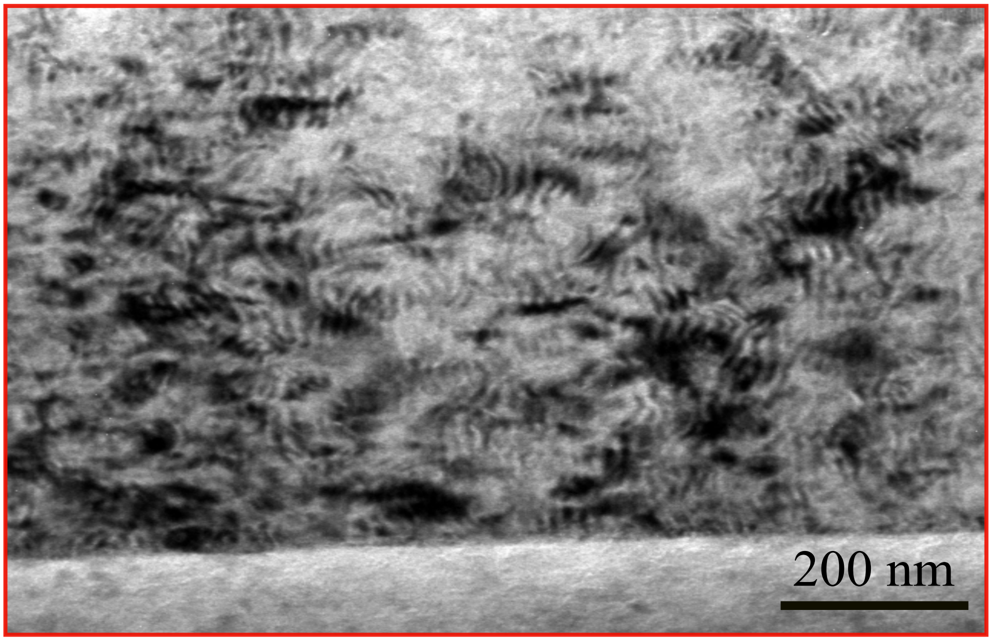

FIG. S1. Dark-field image of ICOM CO phase.

B. Unidirectional charge-stripe pattern in CO phase

Broken spatial symmetry is an integral aspect of CO transition. Further, the broken spatial symmetry in tandem with strong Coulomb interaction (U), leads to the localization of a charge density. These segregate charge-density modulations are often visualized as a stripe patterns in HR-TEM image. Moreover, the relation of such charge stripe patterns with broken spatial symmetries, *e.g.* translational and rotational symmetry have been demonstrated recently [2]. Figure S2(a) shows a raw HR-TEM image of CoO in [112] zone-axis. The corresponding Fourier filtered image is also shown in the Fig.2(a) in the main text. The HR-TEM image shows a charge-stripe pattern. The inherent charge-stripe patterns in a simple CoO is an unusual observation. Therefore, these stripe pattern also serves a direct evidence of CO in CoO. Furthermore, these charge-stripe patterns are associated to the COM (q_1) CO phase. Figure S2(b) shows the Fourier-filtered HR-TEM image of the raw image, providing a sharper view of existing charge stripe pattern in CoO.

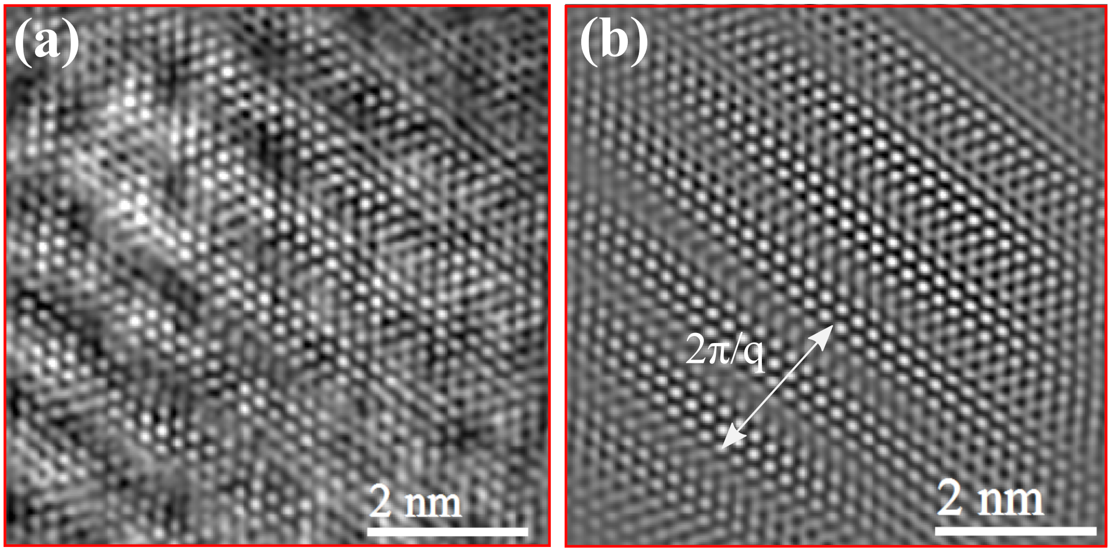

FIG. S2. Unidirectional charge density modulation (charge-stripe) pattern in CoO lattice. (a, b) Raw and Fourier filtered HR-TEM image of CoO, showing the stripe patterns.

C. Disentangled CO phase

We further disentangle the CO phase and host-lattice via Fourier filtering. Figure S3 shows the spatial distribution of disentangled CO, host lattice and the overlapped phase, respectively. Figure S3(a) is HR-TEM image of the CoO lattice, which is retrieved from the COM CO phase. The corresponding FFT is shown in the inset. The host-lattice shows the a regular atomic modulation. However, the COM superlattice shows a localized deviation

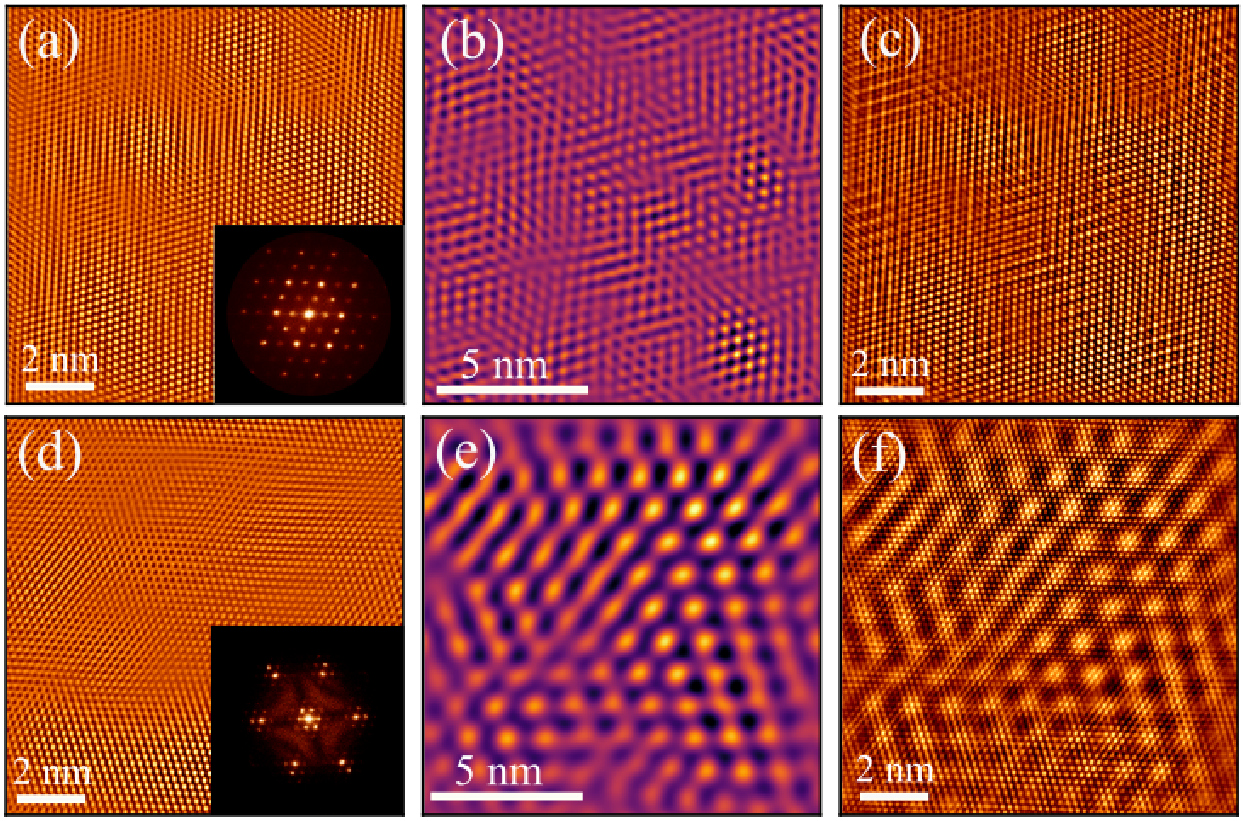

FIG. S3. Disentangling the host-lattice and CO superlattice from the COM and ICOM CO region. (a, d) Host-lattice retrieved from COM and ICOM CO region. (b, e) Real-space image of COM and ICOM superlattice. (c, f) Host-lattice overlapped with the COM and ICOM CO lattice.

in the spatial modulation (Fig.S3(b)). Furthermore, we combine the host-lattice and the COM superlattice to visualize real-space distribution. Figure S3(c) shows a HR-TEM image of CoO, which encompasses the COM superlattice. The combined HR-TEM image (host-lattice + COM CO phase) shows the crossing stripes pattern. The stripe pattern essentially arise due the introduction of COM lattice distortion.

Figure S3(d-f) shows the spatial characteristics of disentangled host-lattice and ICOM CO lattice. The host-lattice and ICOM superlattice shows a regular atomic modulation (Fig.S3(d)). However, the host-lattice overlapped with the ICOM CO phase, shows a unique pattern of a segregated charge-density. Such segregated charge-density patterns is an unprecedented observation in CoO, and indicates an existing rich electronic phase inhomogeneity in CoO lattice. A similar pattern of localized charge-density modulation are often reported in various materials, possessing the charge-density-waves (CDW). These localized charge-density patterns arise due to the stoichiometric disordering ($Co_{1-x}O$) induced CO in CoO (See Fig.2(c) in main text). The simulated HR-TEM image of $Co_{1-x}O$, $x = 4.17\%$, also shows a similar pattern of charge-density modulation (See Fig.S15(2) and Fig.2(d) in main text) and therefore, affirms the nonstoichiometry induced CO in CoO.

D. Spectroscopic investigation of CO in CoO: High resolution electron energy-loss spectroscopy (HR-EELS) investigation

This section explores the spectroscopic features of CO phases. We acquire spatially resolved HR-EELS of Co-$L_{3,2}$ and O-K edge of CoO, respectively.

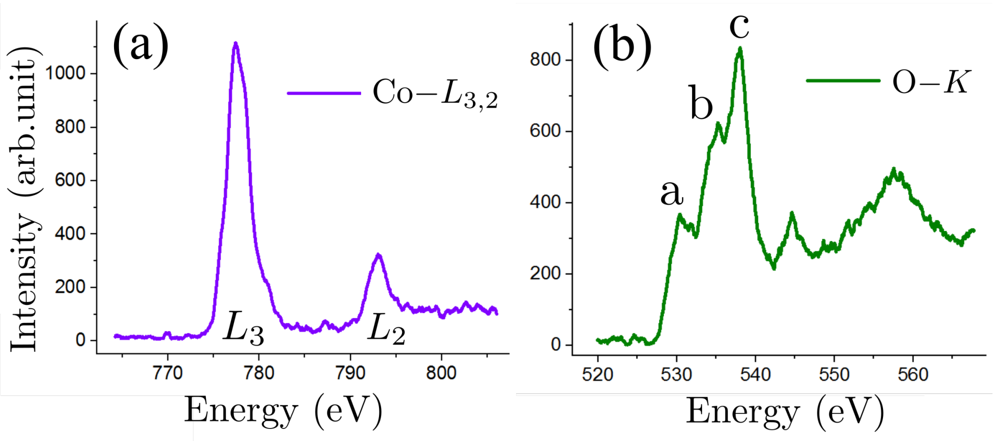

FIG. S4. Electron energy-loss spectra acquired from CoO thin film. (a) Co-$L_{3,2}$ edge (b) O-K edge of CoO.

Co-$L_{3,2}$ Edge:- The Co-$L_{3,2}$ edge of CoO, represents the energy-loss associated to the electronic transition from Co-2$p^{1/2,3/2}$ to unoccupied Co-3$d^{3/2,5/2}$. In stoichiometric CoO, Co-$L_{3,2}$ edge splits in two sharp energy thresholds, delimited by $\sim$ 13 eV (Fig.S4(a)). The energy difference between L_3, and L_2 edge threshold signifies the strength of spin-orbital coupling (SOC) [3]. The intensity ratio of L_3/L_2 edge is sensitive to the atomic charge-state [4]. The shape of L_3 edge peak is highly sensitive to the crystal-field environment *e.g.* tetrahedral (T_d), octahedral (O_h) symmetry. Furthermore, the splitting of L_3 edge peak is a characteristics spectral feature of coexisting mixed-valence charge state, which occurs due to a chemical shift. The splitting in the L_3 edge peak have been measured to probe the mix-valence charge-states in various materials *e.g.* cobaltates, magentites *etc.*, [5].

O-K Edge:- The O-K edge onset of CoO, represents the energy-loss, associated to the electronic transition from O-1s to unoccupied hybridized O-2$p$$-$Co-3$d$ states. The O-K edge of CoO splits in three major characteristics peaks, a, b, c as shown in Figure S4(b). The peak a reflect the transition in the hybridized O-2$p$$-$Co-3$d$ states and the peaks b, c originates from the transitions to unoccupied hybridized O-2$p$$-$Co-4$sp$ electronic states [3]. In pure CoO, the intensity of peak a is comparable to peak b. Moreover, the intensity of O-K pre-edge peak is highly sensitive to hole doping, as reported various oxide materials [6].

Figure S4 shows an experimental Co-$L_{3,2}$ and O-K edge spectra of CoO. The Co-$L_{3,2}$ edge

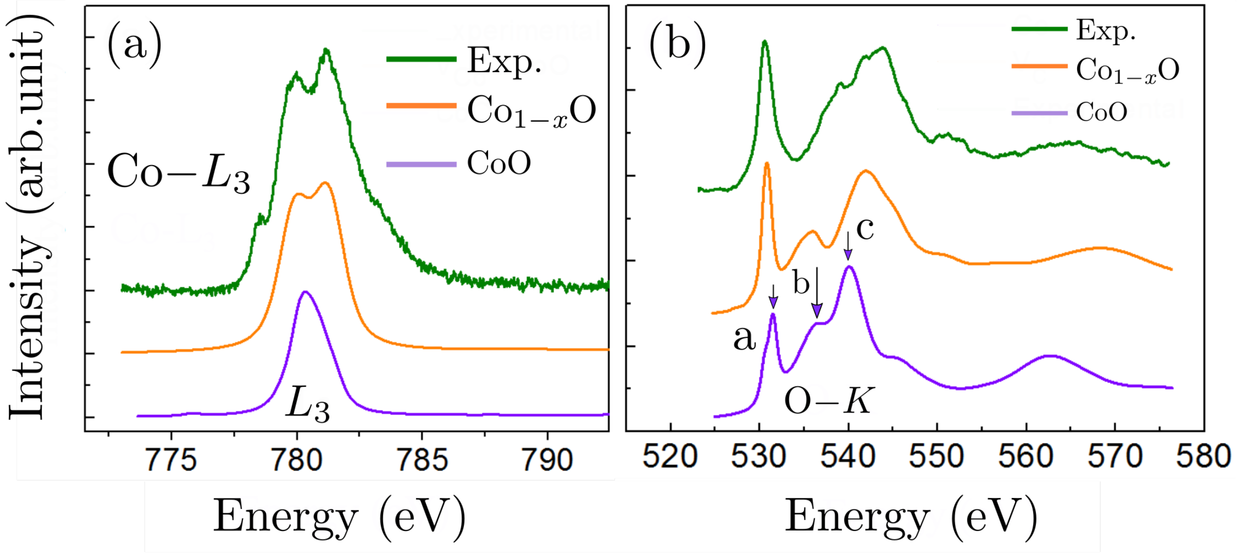

FIG. S5. Comparing the simulated and experimental (a) Co-L_3 and (b) O-K edge of CoO and $Co_{1-x}O$, respectively.

exhibits the characteristic L_3 and L_2 peaks, which are separated by $\sim$ 13 eV. Similarly O-K edge shows the characteristics major a, b, c peaks of CoO. However, the EELS spectra acquired from the CO regions show a distinct spectral features. Figure S5 shows the Co-L_3 and O-K edge, acquired from the CO region of CoO. The Co-L_3 peak from CO phase splits in two fine structures. These fine structures are separated by $\sim$ 1 eV. The splitting of L_3 peak indicates a chemical shift and further hints the possibility of existing mixed-valence charge-states in CoO. We confirm and rationalize such possibility by simulating the Co-L_3 edge of $Co_{1-x}O, x = 4.17\%$. The simulated Co-L_3 edge shows a close proximity with the experimental spectra. The computed Co-L_3 also split in two separated peaks. However, the simulated Co-L_3 edge of stoichiometric CoO shows a single Co-L_3 peak (Fig.S5(a)).

The O-K edge acquired from the CO region, also displays a distinct spectral features. The O-K edge exhibits an unusual intense pre-edge peak, *'a'*. The intensity of peak *'a'* surpasses other peaks, *b*, *c*. The intense peak *'a'* also indicates the abundance of unoccupied Co-3d states, in the thin film. The simulated O-K edge of $Co_{1-x}O, x = 4.17\%$, shows a good agreement with the experimental O-K edge and reveals an intense peak-*'a'*. However, the simulated O-K of pure CoO, shows the usual characteristics peak (Fig. S5(b)).

The simulated Co-$L_{3,2}$ and O-K edge of $Co_{1-x}O$ reveal a close proximity with the experimental Co-$L_{3,2}$ and O-K edge of CO phase in CoO. In $Co_{1-x}O$, with $x = 4.17\%$, the cation deficiency introduces two hole states in the O-p band. These holes are compensated by the charge-transfer process from the V_{Co}-adjacent Co ion via partial charge delocalization of Co-3d states. The resultant charge-transfer also transforms the V_{Co}-adjacent divalent Co^{+2} ion to Co^{+3} ion. These nascent localize trivalent charge-states reflect in between the band-gap of stoichiometric CoO. Moreover, the enhanced ionic charge ($q^{'}_{Co} = 1.50\ e^{-}$) and magnetic

moment ($m' = 3.15\ \mu_B$) on V_{Co} adjacent to the Co atom suggests the presence of localized Co^{+3} state in $Co_{1-x}O$ matrix. Therefore, the DFT calculations and Bader charge analysis also validates the mixed-valence charge-states (Co^{+2}, Co^{+3}) in the $Co_{1-x}O$ matrix (See Section IV, main text). A splitting of the Co-L_3 indicates the presence of such mixed-valence charge states. Consistently, the charge-transfer from Co-3d orbitals enhances the population of unoccupied levels of hybridized Co-3d−O-2p states, above the fermi-level. These abundant unoccupied energy levels are reflected in the intense pre-edge peak *'a'* of O-K edge (Fig.S5(b)). Therefore, the spectral features of Co-$L_{3,2}$ and O-K edge of CO phases suggest the V_{Co}-induced mixed-valence charge states in CoO.

II. CALCULATION METHOD OF COARSE-GRAINED PHASE FIELD

This section describes the calculation procedure for extracting the coarse-grained phase field (ϕ(r)) of order-parameter (OP) (See Section III, main text). We follow the procedure as described in earlier Ref. [7]. We first Fourier filter the region, except the modulation associated to CO modulation vector q_i. The filtered real-space image, associated to modulation q_i, can be approximated as,

$$I(r) \sim sin(q_i.r + \phi(r)) \tag{1}$$

Next we use the phase lock-in method to extract the fluctuation in the OP. The filtered image is first multiplied with two reference signals *i.e,* $sin(q_i.r)$, $cos(q_i.r)$. The resultant reference signals can be represented mathematically as :

$$X(r) = sin(q_i.r) \cdot sin(q_i.r + \phi(r)) \tag{2}$$

$$Y(r) = cos(q_i.r) \cdot sin(q_i.r + \phi(r)) \tag{3}$$

$$X(r) = \frac{1}{2}\left(cos(\phi(r)) - cos(2q_i.r + \phi(r))\right) \tag{4}$$

$$Y(r) = \frac{1}{2}\left(sin(\phi(r)) - sin(2q_i.r + \phi(r))\right) \tag{5}$$

The higher frequencies in the resultant reference signals are further removed by utilizing the low pass filter,

$$\tilde{X}(r) \approx cos(\phi(r)) \tag{6}$$

$$\tilde{Y}(r) \approx sin(\phi(r)) \tag{7}$$

The phase-field ϕ(r), can further be obtained by the following equation,

$$\phi(r) = tan^{-1}\left[\tilde{Y}(r)/\tilde{X}(r)\right] \tag{8}$$

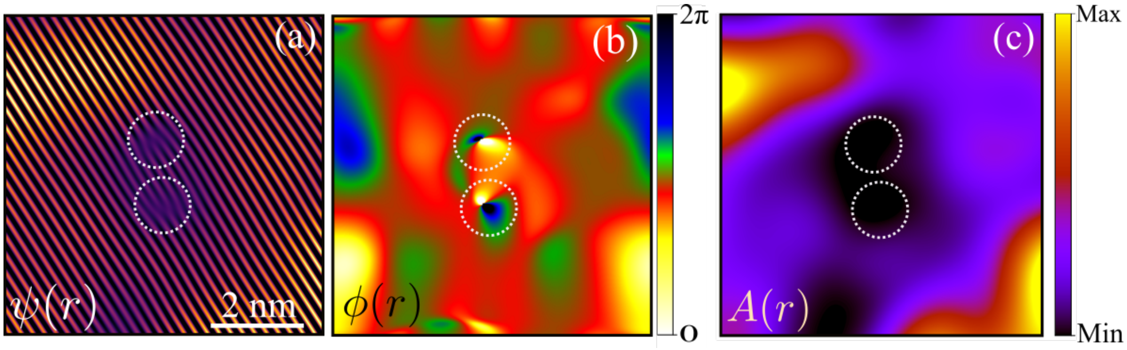

FIG. S6. Example of calculating the spatial distribution of phase and amplitude field of a complex modulation field. (a) Unidirectional density modulation of host-lattice. (b, c) Spatial distribution of corresponding $\phi(r)$ and $A(r)$ field, respectively.

Figure S6(a) represents the OP in the form a density modulation of the host-lattice. The density modulation contains two topological defect at the center (marked by circles). These topological defects are heading in opposite direction from the center. Coherently, the corresponding $\phi(r)$ map reveals the two phase singularities at the location of the topological defect core. The phase also winds 2π across the dislocation core [7]. Consistently, $A(r)$ vanishes at the center of the topological defects (Fig.S6(c)).

Figure S7 compares the spatial distribution of $\phi(r)$ and $A(r)$ of COM CO phase, with

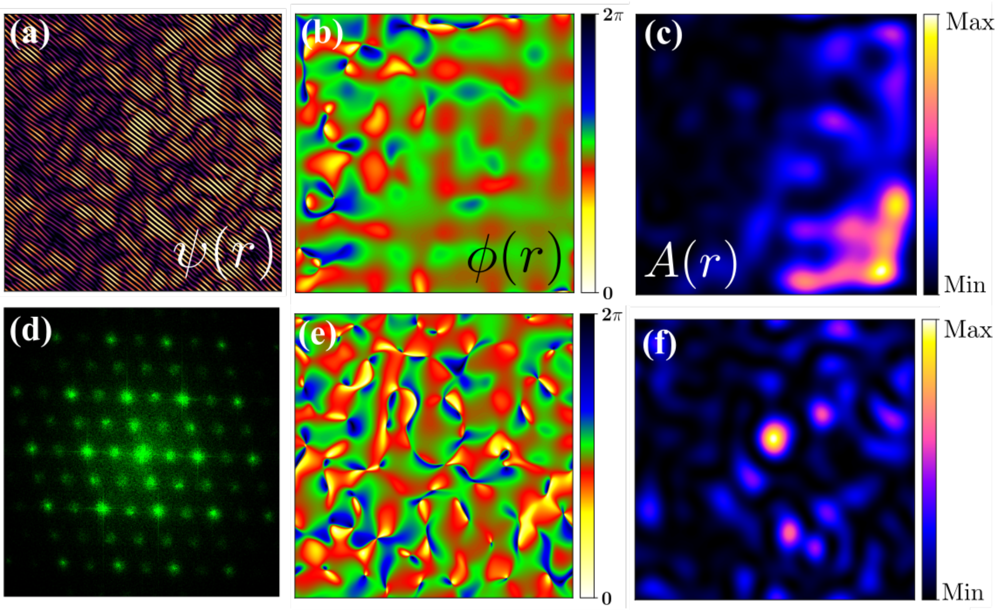

FIG. S7. Coarse grain phase field mapping of COM and CoO lattice. (a) Density modulation of COM CO phase. (b, e) $\phi(r)$ distribution of host-lattice and COM CO phase. (d) Fourier map showing the presence of COM superlattice reflections in the host-lattice of CoO. (c, f) $A(r)$ distribution of host-lattice and COM CO phase.

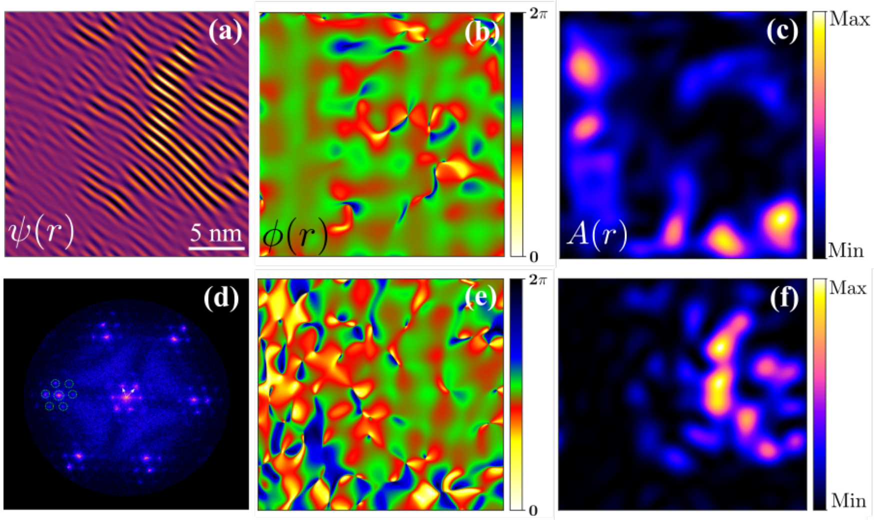

FIG. S8. Coarse grain phase-field mapping of the ICOM phase in CoO. (a) Density modulation of ICOM phase. (b, e) Spatial map of $\phi(r)$, associated to the host-lattice and ICOM CO phase. (d) The EDP showing the ICOM superlattice bragg reflections. (c, f) $A(r)$ distribution of host-lattice and ICOM CO phase.

the host-lattice of CoO. Figure S7(a) represents the the density modulation of COM (q_1) CO phase. The density modulation reveals a large scale topological defects. The spatial fluctuation in $\phi(r)$ and $A(r)$ of native CoO lattice, is shown Figure S7(b,c) respectively. Figure S7(e,f) shows the $\phi(r)$ and $A(r)$ maps of COM CO phase. The $\phi(\mathbf{r})$ shows pronounced phase singularities over a wider region and indicates larger dislocations in COM phase. The maximum intensity of $A(r)$, associated to COM phase is $\sim 3\%$ host-lattice. The intensity of host-lattice maximizes on the spatial location, where the intensity maximum of COM lattice minimizes and vice-versa. The diffuse intensity of COM bragg reflection in reciprocal crystal map, probably reflects the existence of higher phase singularity in $\phi(r)$.

Figure S8 represents the spatial distribution of $\phi(r)$ and $A(r)$, associated to the ICOM lattice distortions. The spatial distribution of the ICOM phase, exhibits few topological

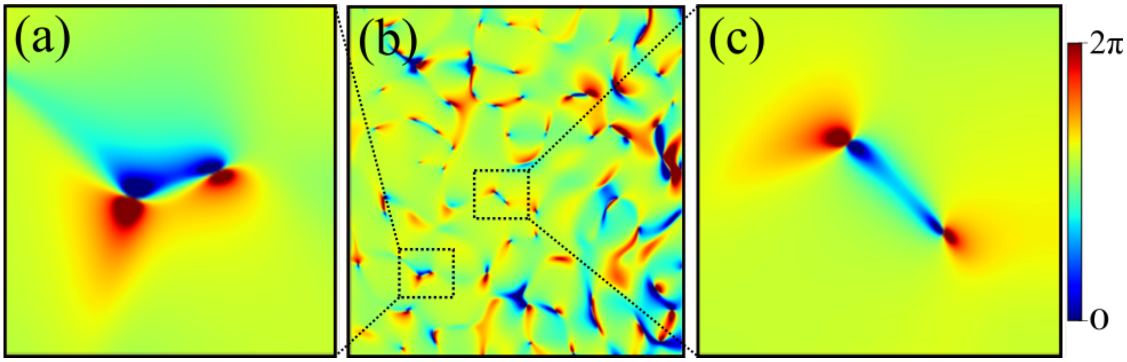

FIG. S9. Connected phase singularities in the $\phi(r)$ map of COM CO phase.

defects (Fig.S8(a)). The $\phi(r)$ and $A(r)$ map of host-lattice is shown in Figure S8(b,c). The corresponding local Fourier map shows the presence of ICOM superlattice reflections. Consistently, the spatial map of $\phi(r)$, exhibits the phase-singularities on the corresponding location of topological defects. Further, the intensity of $A(r)$, associated host-lattice diminishes on the locations, where the intensity of $A(r)$, associated to ICOM phase enhances (Fig.S8(e,f)). The maximum intensity of $A(r)$, associated to the ICOM phase is $\sim$ 57% than the host-lattice. Therefore, the spatial distribution of $A(r)$ suggests that the ICOM CO phase dominates than the COM CO phase in the CoO (See Section III, main text).
Figure S9(a-c) explore the nature of the phase singularities in the COM CO phase. Figure S9(b) shows an interesting nature localized phase singularities. These singularities exhibit a interconnected network.

III. DENSITY FUNCTIONAL THEORY CALCULATIONS

A. Computational methods : WIEN2K

First principles electronic structure calculations were also carried out by using the WIEN2K code. WIEN2K is a full potential linear augmented plane wave plus local orbital based method for density functional theory (DFT) calculations. The AFM-II type magnetic structure of CoO is considered for the calculations. The electronic structure of $Co_{1-x}O$ is simulated by implanting a single Co vacancy (V_{Co}) in the a (2×2×2) supercell of CoO, which contains a total 48 atoms ($Co_{1-x}O, x = 4.17\%$). Resultant distorted structural configuration was fully relaxed with the convergence criteria of charge (1×10^{-3} e^-), energy (1×10^{-4} Ry.), force (1×10^{-3} Ry./a.u.) convergence with the GGA-PBE exchange correlation functional. The relaxed lattice parameters are obtained as $a = 5.88$ Å, $c = 26.99$ Å. Calculations were carried out by separating the valence and core states by -6 Ry. Spin-polarized self-consistent field (SCF) cycle calculations were carried out with taking 3000 k points in the first Brillouin zone. The $R_{MT}.K_{max}$ parameter was set to 7. In order to carry out the band-gap correction, calculations were performed with the modified Becke-Johnson (mBJ) method. The WIEN2K calculations are majorly used to simulate the ELNES spectra of Co-$L_{3,2}$ and O-K edge, respectively.

B. Crystal structure

The details of the computational method using VASP code can be found in the Method section of main text. The hexagonal unit-cell approach was utilized to simulate the $Co_{1-x}O$. Figure S10 represents the electronic structure of stoichiometric CoO. CoO is AFM-II antiferromagnetic insulator. In CoO the magnetic moments stacks parallel in (111) crystal plane. The electronic structure of non-stoichiometric CoO ($Co_{1-x}O$) is simulated by implanting

a V_{Co} in a (2×2×2) supercell of CoO, containing total 48 atoms ($Co_{1-x}O, x = 4.17\%$) (Fig.S10(b).

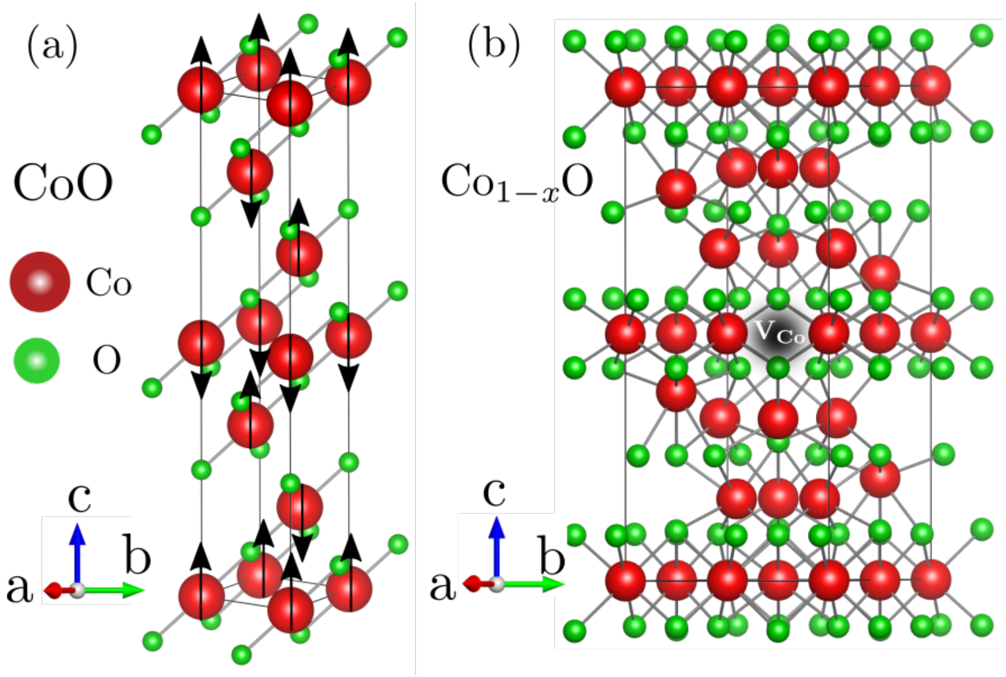

FIG. S10. Stoichiometric and non-stoichiometric electronic structures of CoO, considered for the density functional theory calculations. The electronic structure of (a) Stoichiometric and (b) Non-stoichiometric ($Co_{1-x}O, x = 4.17\%$) CoO.

The computed Co−O bond-length in pure CoO, is found to be 2.13 Å. However, the embedded V_{Co} in $Co_{1-x}O$, profoundly influences the electronic properties at V_{Co} adjacent atomic sites. The Co−O bond-length on V_{Co} adjacent site decreases from 2.13 Å to 1.97 Å. The V_{Co} adjacent O atom moves towards the nearest Co ion. However, far away from the V_{Co}, the bond-length is marginally re-arrange less then $\sim$ 0.01 Å. The electronic structure properties of cation deficient CoO ($Co_{1-x}O$) is also investigated previously [8–11].

C. Defect induced charge-state transition in $Co_{1-x}O$

In this section, we explore the influence of V_{Co} on the Co atoms, located in the first and second coordination shell (1CS, 2CS), from V_{Co}, respectively. The total DOS profile of CoO and $Co_{1-x}O$ ($x = 4.17\%$) can be found in the main text (See Section IV, main text). The ramification of V_{Co} in $Co_{1-x}O$, is a distortion of the octahedral crystal-field (Δ_{oct}). The symmetry reduction in octahedra (O_h) substantially influence the electronic properties of V_{Co} adjacent Co and O atoms, respectively. Therefore, we explore the electronic nature V_{Co} adjacent Co atom in $Co_{1-x}O$.

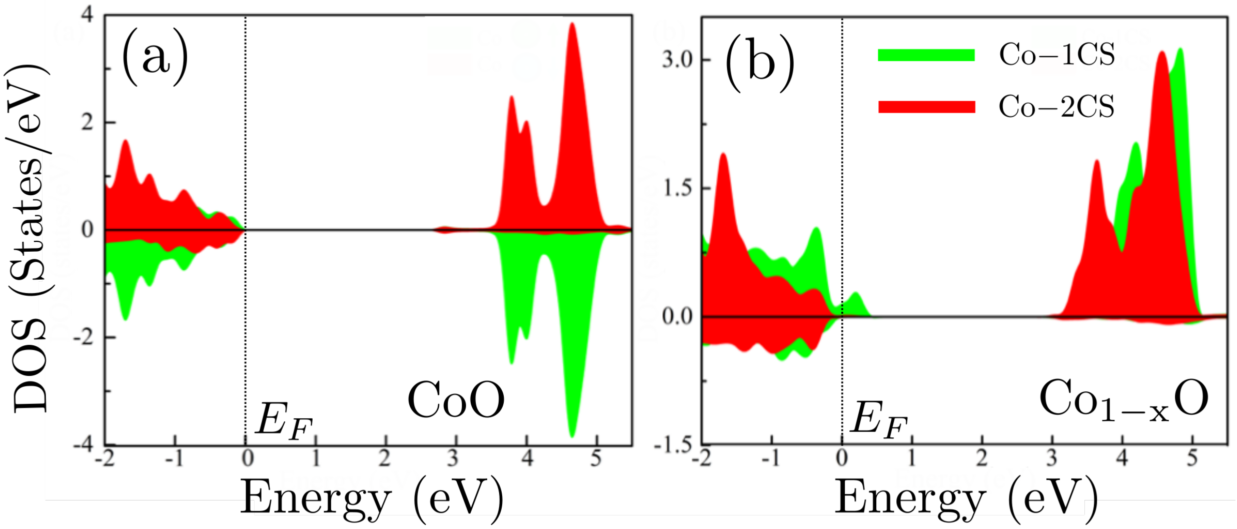

FIG. S11. (a) DOS profile of Co-d orbitals in stoichiometric CoO. (b) DOS profile of Co-d orbitals located in the 1CS and 2CS from V_{Co}.

Figure S11(a) shows the DOS profile of Co-3d states of stoichiometric CoO. The complementary populated spin-up and spin-dn channel reflects the antiferromagnetic arrangement of the Co spin magnetic moment. However, the DOS profile of Co atoms $Co_{1-x}O$, exhibits a distinct distribution (Fig.S11(b)). The DOS cross the Fermi-level (E_F) and exhibits the metallic nature. However, these additional states in $Co_{1-x}O$, are only contributed by V_{Co} adjacent Co atoms (1CS). The DOS of Co-3d in 2CS is similar to the Co-3d of pure CoO. The enhances Co-3d state at E_F also shows strong hybridization with O-2p states. Therefore, the effect of V_{Co} in $Co_{1-x}O$, is to introduce an addition energy states in the band-gap. Furthermore, these nascent orbital states are solely contributed by V_{Co} adjacent hybridized Co-3d−O-2p states. These rearranged energy states also indicates a pronounced charge-transfer in the vicinity of V_{Co} in $Co_{1-x}O$ (See Section IV (Fig.4), main text).

Next, we explore the DOS profile of the degenerated orbitals of Co-d $i.e,$ t_{2g} and e_g orbtials, of CoO and $Co_{1-x}O$, respectively (Fig.S12). For stoichiometric CoO, the DOS profile of the degenerated t_{2g} and e_g orbitals show a symmetric distribution (Fig.S12(a)). The symmetric nature of DOS reflects the AFM arrangement of spins. In stoichiometric CoO, the Co lies in the high-spin state (HS $[(\uparrow 3\,t_{2g} \downarrow 1\,t_{2g})\,(\uparrow 2\,e_g)[S=2]]$). The t_{2g} DOS dominates at E_F, due the abundance of electron density. The t_{2g} states also contributes majorly on the top and bottom of valence and conduction band, respectively. Figure S12(b) indicates a rearrangement in the t_{2g} and e_g orbital states of $Co_{1-x}O$. The DOS profile shows that, the t_{2g} states now also dominates beyond the E_F, which further signifies that the energy of the t_{2g} states has increased. The orbital-redistribution also highlights the charge-

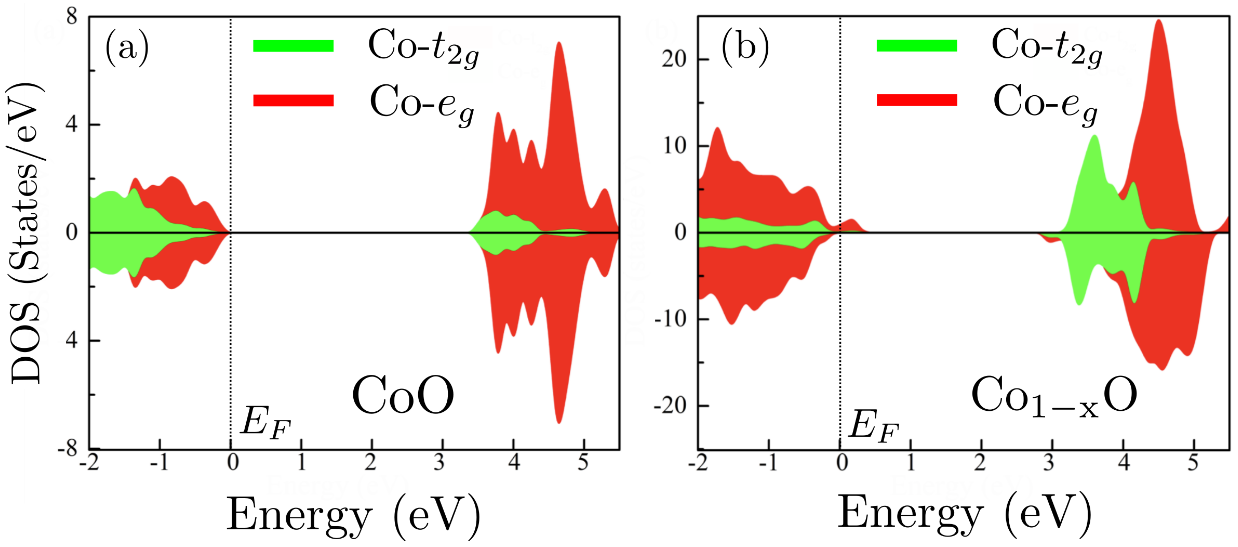

FIG. S12. Orbital-decomposed spin-polarized DOS of degenerated t_{2g} and e_g orbitals of Co-3d states for (a) stoichiometric CoO and (b) Co$_{1-x}$O , $x = 4.17\%$.

transfer in Co$_{1-x}$O. Moreover, the electrons only in the spin-up channel of t_{2g} orbitals, cross the E_F. The corresponding DOS of the partial orbitals can be found in the Section IV (Fig.4) of main text. These undirectional spin-states above the E_F are commonly referred as half-metallic states. Cation deficiency induced HM states are also reported in various transition-metal-mono-oxides *e.g.,* CaO, MnO, and NiO [10].

D. Defect driven Jahn-Teller distortion in Co$_{1-x}$O

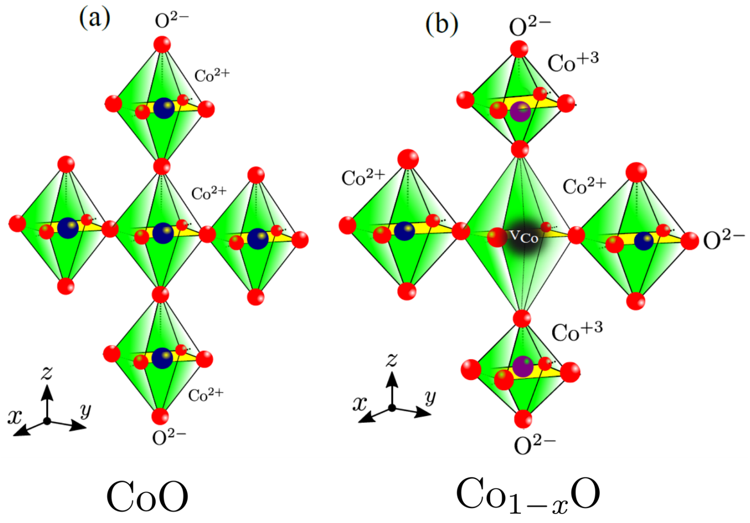

FIG. S13. Cation order imperfection-driven Jahn-Teller distortion in CoO. (a) Octahedral ligand-field of CoO. (b) Octahedral distortion in Co$_{1-x}$O

This section describes the O_h geometric distortion in $Co_{1-x}O$, which eventually leads to a Jahn-Teller distortion. Figure S13 displays the O_h electronic environment of the CoO and $Co_{1-x}O$, respectively. The stoichiometric CoO shows a regular octahedral ligand-field environment. However, a cation deficiency profoundly distorts the local ligand-field and leads to a JT displacement. In the JT displacement the defected O_h elongates vertically ∼0.16 Å and concomitantly stretch the adjacent O_h in the lateral direction (Fig. S13(b)). The JT distortion facilitates the hole-compensation process in the vertically opposite O_h. The hole compensation introduces two localized Co^{+3} states, and thus leads to a charge disproportion in $Co_{1-x}O$. Furthermore, the local structural modification destabilizes the crystal-field and induces a half-metallic state (See Section IV, main text.)

E. Band-structure calculation

In this section, we show the computed band-structure of CoO and $Co_{1-x}O$, respectively (Fig.S14). The computed band-structures are coherent with the total DOS (See Section IV (Fig.4(a,b)), main text. For stoichiometric CoO, the valence band maximum and conduction band minimum are delimited by $\sim$ 2.78 eV at Γ point. This energy difference (ΔE) represents the band gap of stoichiometric CoO. However, for $Co_{1-x}O$ a significant redistribution in the band-states can be noticed. The band-states largely enhances in $Co_{1-x}O$. Moreover, few additional band-states beyond the E_F can be distinguish. These additional band-state are solely contributed by V_{Co} adjacent hybridized Co-3d−O-2p states (Section III C). Therefore, the influence of V_{Co} in $Co_{1-x}O$ is to introduce additional band-states in the band-gap of CoO. These localized band-state essentially represents the Co^{+3} states at V_{Co} adjacent Co atomic site (See Section IV, main text).

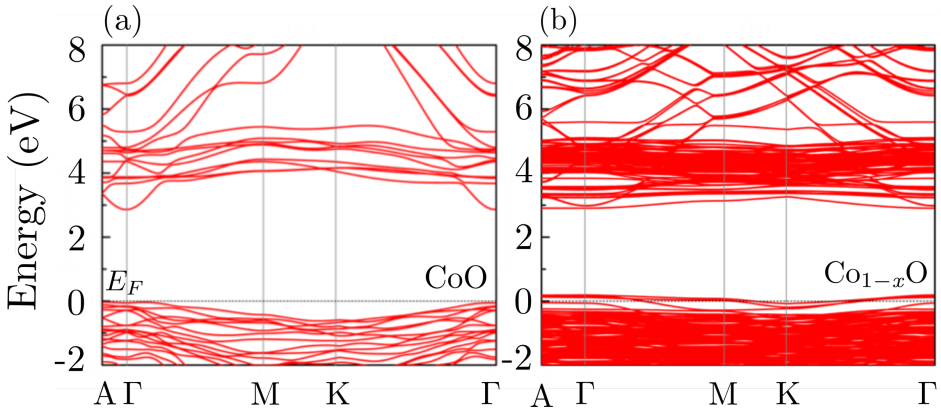

FIG. S14. The computed electronic band structures of (a) stoichiometric CoO and (b) $Co_{1-x}O$. In the band structure of the $Co_{1-x}O$, the additional metallic states along the dispersion channel Γ, represent the localized Co^{+3} state.

IV. QUANTITATIVE COMPARISON OF EXPERIMENTAL AND SIMULATED HR-TEM

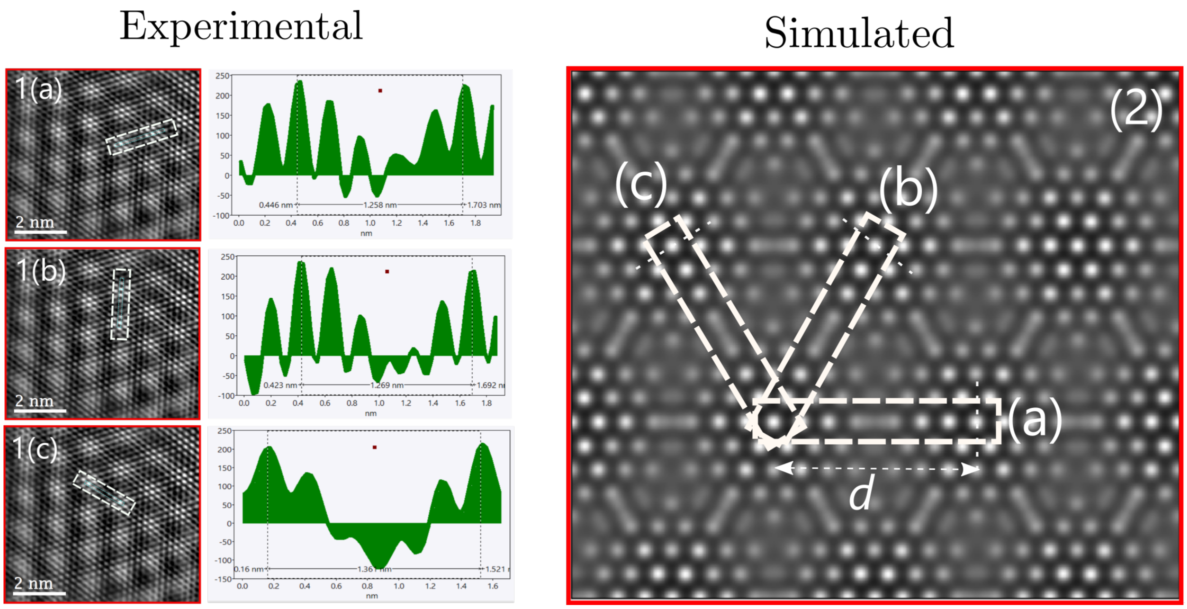

FIG. S15. Quantitative comparison of experimental and simulated HR-TEM image of CoO. 1(a-c) Experimental TEM image showing the line scan in three different directions. (2) Simulated HR-TEM image of $Co_{1-x}O$ showing the line scan along the directions shown in 1(a-c).

In the figure S15(1(a-c)), we analyze the experimental image (7.44 nm $\times$ 6.56 nm). We focus on a particular section in the image and measure the inter-atomic distance between the most probable intense atoms in the neighboring center of segregated density. The figure S15(1(a-c)) shows the line scan and corresponding distance along the three directions. The average interatomic distance is $\sim$ 1.29 nm. Further, in the figure S15(2), we compare the simulated image section of cation deficient CoO with the experimental image section. The interatomic distance d in the simulated image found $\sim$ 1.23 nm along different directions. The distance d is nearly similar to the experimental distances in the figure S15(1(a-c)). The quantitative similarity between the experimental and simulated image is helpful to visualize the most probable effect of a cation disordering in the electron micrographs (See Section II(Fig.2), main text).

Multislice image simulation parameters

1. Acceleration voltage = 300 kV

2. C_s = -0.030 mm

3. C_c = 1.00 nm

4. Δf = -9.00 nm

5. C_5 = 5.00 mm

6. α = 0.15 mrad

7. Project Potential = Weickenmeier

8. Sample thickness = 2.77 nm

References

[1] C. Chen and S.-W. Cheong, "Commensurate to incommensurate charge ordering and its real-space images in $La_{0.5}Ca_{0.5}MnO_3$," *Physical Review Letters*, vol. 76, no. 21, p. 4042, 1996.

[2] R. Comin, R. Sutarto, E. da Silva Neto, L. Chauviere, R. Liang, W. Hardy, D. Bonn, F. He, G. Sawatzky, and A. Damascelli, "Broken translational and rotational symmetry via charge stripe order in underdoped $YBa_2Cu_3O_{6+y}$," *Science*, vol. 347, no. 6228, pp. 1335–1339, 2015.

[3] Y. Zhao, T. E. Feltes, J. R. Regalbuto, R. J. Meyer, and R. F. Klie, "In situ electron energy loss spectroscopy study of metallic co and co oxides," *Journal of Applied Physics*, vol. 108, no. 6, p. 063704, 2010.

[4] Z. Wang, J. Yin, and Y. Jiang, "Electron energy-loss analysis of cation valence states and oxygen vacancies in magnetic oxides," *Micron*, vol. 31, no. 5, pp. 571–580, 2000.

[5] H. Tan, S. Turner, E. Yücelen, J. Verbeeck, and G. Van Tendeloo, "2d atomic mapping of oxidation states in transition metal oxides by scanning transmission electron microscopy and electron energy-loss spectroscopy," *Physical review letters*, vol. 107, no. 10, p. 107602, 2011.

[6] N. Gauquelin, D. Hawthorn, G. Sawatzky, R. Liang, D. Bonn, W. Hardy, and G. Botton, "Atomic scale real-space mapping of holes in $YBa_2Cu_3O_{6+\delta}$," *Nature communications*, vol. 5, p. 4275, 2014.

[7] B. H. Savitzky, I. El Baggari, A. S. Admasu, J. Kim, S.-W. Cheong, R. Hovden, and L. F. Kourkoutis, "Bending and breaking of stripes in a charge ordered manganite," *Nature communications*, vol. 8, no. 1, p. 1883, 2017.

[8] U. Wdowik and K. Parlinski, "Electronic structure of cation-deficient CoO from first principles," *Physical Review B*, vol. 77, no. 11, p. 115110, 2008.

[9] P. Ignatiev, N. Negulyaev, D. Bazhanov, and V. Stepanyuk, "Doping of cobalt oxide with transition metal impurities: Ab initio study," *Physical Review B*, vol. 81, no. 23, p. 235123, 2010.

[10] D. S. Negi, R. Datta, and J. Rusz, "Defect driven spin state transition and the existence of half-metallicity in CoO," *Journal of Physics: Condensed Matter*, 2019.

[11] D. S. Negi, D. Singh, P. A. van Aken, and R. Ahuja, “Spin-entropy induced thermopower and spin-blockade effect in CoO,” *Physical Review B*, vol. 100, no. 14, p. 144108, 2019.
